# The siRNA-mediated knockdown of AP-1 restores the function of the pulmonary artery and the right ventricle by reducing perivascular and interstitial fibrosis and key molecular players in cardiopulmonary disease

**DOI:** 10.1186/s12967-024-04933-1

**Published:** 2024-02-05

**Authors:** Ioana Karla Comarița, Gabriela Tanko, Iliuță Laurențiu Anghelache, Adriana Georgescu

**Affiliations:** 1 Institute of Cellular Biology and Pathology ‘Nicolae Simionescu’ of Romanian Academy, Bucharest, Romania; 2 ‘Victor Babeș’ National Institute of Pathology, Bucharest, Romania

**Keywords:** Cardiopulmonary disease, Pulmonary arterial hypertension, Cardiac and pulmonary fibrosis, Monocrotaline, siRNA AP-1

## Abstract

**Background:**

Pulmonary hypertension (PH) is a complex multifactorial vascular pathology characterized by an increased pulmonary arterial pressure, vasoconstriction, remodelling of the pulmonary vasculature, thrombosis in situ and inflammation associated with right-side heart failure. Herein, we explored the potential beneficial effects of treatment with siRNA AP-1 on pulmonary arterial hypertension (PAH), right ventricular dysfunction along with perivascular and interstitial fibrosis in pulmonary artery-PA, right ventricle-RV and lung in an experimental animal model of monocrotaline (MCT)-induced PAH.

**Methods:**

Golden Syrian hamsters were divided into: (1) C group-healthy animals taken as control; (2) MCT group obtained by a single subcutaneous injection of 60 mg/kg MCT at the beginning of the experiment; (3) MCT-siRNA AP-1 group received a one-time subcutaneous dose of MCT and subcutaneous injections containing 100 nM siRNA AP-1, every two weeks. All animal groups received water and standard chow ad libitum for 12 weeks.

**Results:**

In comparison with the MCT group, siRNA AP-1 treatment had significant beneficial effects on investigated tissues contributing to: (1) a reduction in TGF-β1/ET-1/IL-1β/TNF-α plasma concentrations; (2) a reduced level of cytosolic ROS production in PA, RV and lung and notable improvements regarding the ultrastructure of these tissues; a decrease of inflammatory and fibrotic marker expressions in PA (COL1A/Fibronectin/Vimentin/α-SMA/CTGF/Calponin/MMP-9), RV and lung (COL1A/CTGF/Fibronectin/α-SMA/F-actin/OB-cadherin) and an increase of endothelial marker expressions (CD31/VE-cadherin) in PA; (4) structural and functional recoveries of the PA [reduced Vel, restored vascular reactivity (NA contraction, ACh relaxation)] and RV (enlarged internal cavity diameter in diastole, increased TAPSE and PRVOFs) associated with a decrease in systolic and diastolic blood pressure, and heart rate; (5) a reduced protein expression profile of AP-1S3/ pFAK/FAK/pERK/ERK and a significant decrease in the expression levels of miRNA-145, miRNA-210, miRNA-21, and miRNA-214 along with an increase of miRNA-124 and miRNA-204.

**Conclusions:**

The siRNA AP-1-based therapy led to an improvement of pulmonary arterial and right ventricular function accompanied by a regression of perivascular and interstitial fibrosis in PA, RV and lung and a down-regulation of key inflammatory and fibrotic markers in MCT-treated hamsters.

## Introduction

Pulmonary hypertension (PH) is a complex multifactorial vascular pathology that compresses a multitude of conditions characterized by a mean pulmonary arterial pressure ≥ 25 mmHg at rest, vasoconstriction, remodelling of the pulmonary vasculature, thrombosis in situ and inflammation associated with right-side heart failure ranging from immunosufficiencies syndromes to autoimmune and haematological disorders. The World Health Organization classifies PH into five distinct groups (5th Word Symposium on PH, Niece, 2013) depending on the histopathological and hemodynamic characteristics, etiology and therapeutic approaches. Group 1 refers to pulmonary arterial hypertension (PAH) a heterogeneous syndrome that originates in the small pulmonary arteries which is currently being extensively researched in terms of designing new and highly affective therapeutic approaches but also elucidating the pathobiology underlying this condition. The most common pathophysiological features of PAH disease include: endothelium dysfunction, medial hypertrophy, adventitial hyperplasia of small pulmonary arteries, perivascular inflammation, plexiform lesions formed by the concentric arrangement of endothelial cells with subadjacent myofibroblasts, muscularization in normally nonmuscular arteries, excessive proliferation and impaired apoptosis of vascular cells, vascular remodelling involving complete obliteration of the vessel leading to increased vascular resistance that results in increased right ventricle afterload, hypertrophy and failure [1–6].

Although remarkable progress has been made in the last decade and numerous innovative therapies based on 30 pharmacological agents [7] have been reported to have beneficial effects on the pathological changes induced by the monocrotaline (MCT) administration, the late diagnosis of the patients in clinics together with the delayed administration of a therapy are responsible for the poor prognosis of this disease but also for the way the patients respond to the treatment. For this reason, many studies have reported that initiating a therapeutic intervention in the early stages of the disease would have a significantly higher success rate in terms of hemodynamics, histopathological effects, and prognosis [8]. In this current study, the hamster was used for the first as an MCT-induced PAH murine model, to not only investigate the beneficial effects of treatment with siRNA AP-1 during the early stages of PAH (on fibrosis, vascular remodelling, right ventricular hypertrophy, and hemodynamics), but also to try to provide answers about the molecular mechanisms underlying this pathology. Activator protein 1 (AP-1) is a crucial transcription factor involved in the regulation of a multitude of cellular processes including proliferation, differentiation, cell migration, apoptosis, and survival [9, 10] in response to a variety of extracellular stimuli including cytokines, chemokines, growth factors, and stress signals (reactive oxygen species). This transcription complex has been shown to play an important role in a variety of inflammatory illnesses [11], as well as cancer [12], pulmonary fibrosis [13, 14], atherosclerosis [15], and other cardiovascular diseases [16, 17], by modulating gene expression involved in inflammation and immunity. In recent years, numerous preclinical and clinical therapeutic strategies with very promising outcomes based on AP-1 inhibitors have been established, such as the development of small molecules or natural compounds specifically targeted for the prevention of inflammatory diseases, in particular [18–21]. Blocking the AP-1 transcription complex represents an attractive molecular approach, so this study proposes the use of a targeted siRNA for the silencing of AP-1 in order to restore structural and functional changes in MCT-induced PAH and underlying associated fibrotic and inflammatory processes.

Small interfering RNA (siRNA) is a double-stranded RNA molecule whose mechanism of action is based on post-transcriptional gene silencing. In recent years, siRNA-based therapy emerged as a promising therapeutic platform for development of personalized treatments for a variety of diseases acting in a sequence-specific manner by mRNA degradation for modulating targeted gene expression. There are numerous delivery technologies of siRNA (lipidoid nanoparticles, exosomes, peptides, etc.) developed in order to maximize the treatment efficiency, biosafety, and stability [22–24]. In one of our recent studies, we used a therapeutic strategy based on siRNA Smad2/3 and extracellular vesicles (EVs) transfected with siRNA Smad2/3 in order to restore vascular dysfunction in an hiperlipidemic-hypertensiv (HH) hamster model which mimics human atherosclerotic disease [25]. The results showed that both naked siRNA Smad2/3 and EVs transfected with siRNA Smad2/3 had similar therapeutic effects. In this study we chose to evaluate the naked siRNA AP-1 administration in order to see its therapeutic efficacy upon MCT-induced PAH but also close monitoring of any possible side effects.

## Materials and methods

### Experimental murine model with MCT-induced cardiopulmonary disease (pulmonary hypertension, and associated perivascular/pulmonary fibrosis and right ventricular hypertrophy) injected or not with siRNA AP-1

**Fig. 1:**
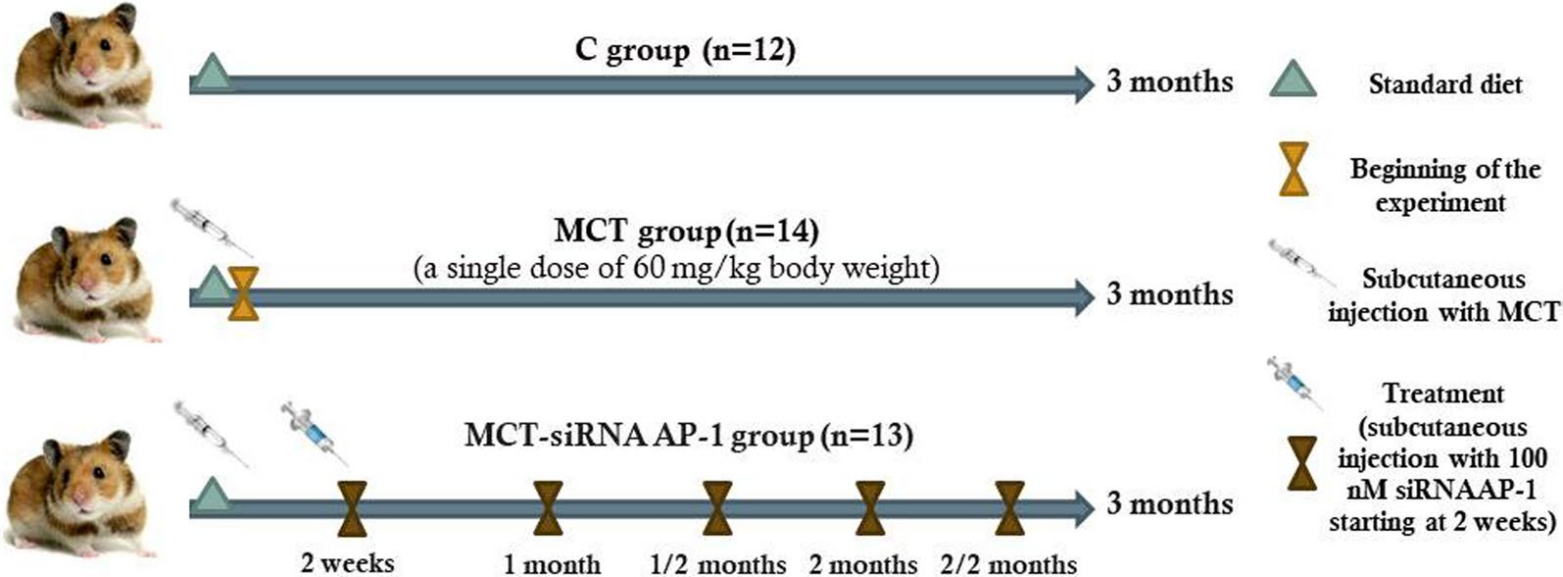
Schematic illustration of experimental animal models obtained for a period of 12 weeks. Golden Syrian hamsters (39 males, 3 months old) were randomly divided into three experimental groups, including: (1) C group, healthy animals; (2) MCT group obtained by a single subcutaneous injection dose of 60 mg/kg monocrotaline (MCT) at the beginning of the experiment (day 0); (3) MCT-siRNA AP-1 group that received subcutaneous injections of 100 nM siRNA AP-1 every two weeks. All animal groups received water and standard chow ad libitum

A total of thirty-nine male Golden Syrian hamsters, approx. 3 months of age, with a body weight of 100–130 g, were randomly assigned into three experimental groups, including: normal healthy animals for control group (C) (n = 12); MCT group (n = 14) that received a single subcutaneous injection of 60 mg/kg body weight MCT at the beginning of the experiment (day 0), in order to induce pulmonary arterial hypertension (PAH) with subsequent right ventricular hypertrophy; MCT-siRNA AP-1 group (n = 13), which 2 weeks after the first injection with MCT, received treatment with siRNA AP-1 (five doses with a concentration of 100 nM each in a volume of 300 µl of phosphate-buffered saline (PBS)), administered subcutaneously every two weeks until the end of the 3-month experimental period (Fig. 1). The AP1S3 siRNA (m): sc-141142 (SantaCruz Biotechnology), designed specifically for the inhibition of AP1S3 expression, was administrated by subcutaneous injection as described by the research in the field [26, 27]. Previous animal studies (performed on mice or rats) indicated that a high dosage of 80 mg/kg of MCT was fatal in the first 3 weeks [28] so we chose to administer a dose of 60 mg/kg MCT to our animal model. This selected concentration was sufficient to generate the animal model with pulmonary arterial hypertension developed over an extended period of time, reflecting the chronic pathological processes present in sick human subjects as well. The aqueous solution of MCT was prepared according to the protocol described in 1967 by Hayashi et al. [29]: MCT (170 mg, Sigma-Aldrich) was dissolved in 1.2 ml of 1 N HCl solution followed by addition of 3 ml distilled water, pH was adjusted to 7.4 using 1 M NaOH solution, and later distilled water was added up to a final volume of 12 ml.

During the entire experimental period of 3 months, all three experimental groups of hamsters were kept in the same housing conditions with a 12/12 h light/dark cycle, a temperature of 25 °C, humidity of 55%, were fed with standard chow containing basal 1%NaCl and received free access to tap water. Body weight was measured at the beginning of the experiment to adjust the treatment doses accordingly and at the end of the 3 months (12 weeks) of experiment, when the hamsters were lightly anesthetized with 2% isoflurane for blood collection. Subsequently, they were sacrificed under intraperitoneal anaesthesia [a solution containing 80 mg ketamine, 10 mg xylazine, 2 mg acepromazine/kg body weight in a sterile isotonic saline (0.9% saline)], perfused with PBS containing 1 mM CaCl_2_ for tissues blood removal in order to collect the organs of interest (pulmonary artery, lung, right ventricle) for weighing the heart and biochemical, structural and functional assays. The experimental protocols were approved by the Ethics Committee from Institute of Cellular Biology and Pathology “Nicolae Simionescu” according to Decision no.11/08.08.2017 and National Sanitary Veterinary and Food Safety Authority (Bucharest, Romania) in compliance with Project Authorization no. 575/13.11.2020. Also, all the animal procedures were carried out in strict accordance with the Guide of the Care and Use of Laboratory Animals published by the US National Institutes of Health (NIH publication no. 85-23, revised 1996), and were conducted in accordance with National, European and International legislation on the use of experimental animals in biomedical research. All surgical procedures were performed under anaesthesia (mild or total) without causing the animals suffering.

### Biochemical analysis of plasmatic parameters

Blood samples were taken from all experimental animal groups by collecting approximately 1 ml of venous blood from the retro-orbital plexus in vacutainers containing ethylenediaminetetraacetic acid (EDTA) solution and centrifuged at 2500 ×*g* for 10 min at 4 °C to obtain plasma. For this, animals were lightly sedated via inhalation with 2% isoflurane (ISOFLUTEK 1000 mg/g) mixed with oxygen under fasting conditions. The procedure was performed at 4 weeks and at the end of the 3-month experimental period. Plasma levels assessing the lipid profile (total cholesterol, HDL-cholesterol, LDL-cholesterol, triglyceride), glucose and hepatic transaminases (ALT-*Alanine Aminotransferase*, AST-*Aspartate Aminotransferase*), were determined by a colorimetric/kinetic method using commercially available kits from DIALAB GmbH, Vienna, Austria. The samples were measured in duplicate using a spectrophotometric assay at a wavelength between 340 and 500/600 nm (Tecan Infinite M200 PRO).

### Non-invasive measurement of haemodynamic parameters

Blood pressure in hamsters was measured with the ADInstruments ML125NIBP (Non-Invasive Blood Pressure) Controller connected to a PowerLab 4/26 system, using specialized tail cuffs. The advantage of this technique is that it provides an accurate recording of pulse rate and blood pressure in the caudal artery in real time. The LabChart software allows the opening of two channels, one for the pulse signal (BPM) and the other for pressure (mmHg), for a typical recording of these parameters. As the cuff inflates, the blood flow in the tail is obstructed and the measurement of the systolic arterial pressure is performed at the moment of the appearance of the first noise determined by the pressure of the cuff. The last noise picked up by the transducer before total deflation at the cuff corresponds to the diastolic blood pressure. Before starting the measurements, the animals were immobilized with the help of a special cage and kept in the dark for 30 min. Every measurement was repeated 8–10 times for the reproducibility of the results. Five animals from each group were evaluated.

### Echocardiographic assessments

Transthoracic echocardiography was performed at the end of the 12 experimental weeks. The high-resolution ultrasonic imaging system for small animals Vevo 2100 equipment (VisualSonics Inc., Toronto, ON, Canada) with MS 250 transducer (12–24 MHz) was used for this diagnostic procedure. The animals have been prepared in advance by removing the fur from the chest with a hair clipper so as not to interfere with the signal of the device. Throughout the procedure, hamsters were lightly anesthetized with 2% inhaled isoflurane in combination with oxygen and placed on a heated platform to maintain a constant body temperature. Vital signs (heart rate, pulse) were constantly monitored. Through this analysis, parasternal sections were obtained both on the long and the short axis to determine the structure and function of the right ventricle and the pulmonary artery. B mode (two-dimensional), M mode were used to assess anatomical and functional characteristics and Pulsed Wave (PW) Doppler mode was used to measure the hemodynamic characteristics of blood flow. The right side of the heart was investigated in: (1) parasternal long axis view, in order to measure pulmonary artery (PA) diameter, right ventricle wall thickness (RVWT) at the level of proximal right ventricle (PRRV), proximal ventricle outflow chamber during systole and diastole (PRVOFd, PRVOFs) and pulsed waved doppler (PW) for PA peak flow velocity (PFV) and velocity time integral (VTI); (2) parasternal short axis view for PA PFV and VTI measurement; and (3) in apical four chamber were measured RV systolic and diastolic area and tricuspid annular plane systolic excursion (TAPSE). VevoLab300 software was used to process and analyse images obtained from all groups of animals. Five animals from each group were followed.

Hamster echocardiography.

### Myographic analysis of functional responses of isolated pulmonary arteries

In order to see the response capacity of the pulmonary artery to contract and relax after MCT injection or siRNA AP-1 treatment and to analyse vascular dysfunction, the myographic system (Multi Myograph System-model 620 M in combination with the Automatic Buffer Filler System-625FS—Danish Myo Technology, DMT), a device that investigates the vascular reactivity, was used. The isolated arterial fragments (150–200 μm in diameter) were placed on an elastomer to remove any lipid residue and cut under a microscope to a length of 2 mm. They were then mounted in the wire-myograph chamber and immersed in HEPES sodium salt buffer, pH = 7.45 at 37 °C. Subsequently, ACh (acetylcholine) was used to induce vessel relaxation and NA (noradrenaline) to induce vessel contraction. Before recordings starts, the calibration of the optimal operating diameter was performed and the standard start procedure was checked for the integrity of the mounted blood vessel and the presence of the intact endothelium by stimulation with 3 × 10^–7^ M NA. Oxygen bubbling in the myograph’s organ chamber was constant throughout the experiment to maintain optimal blood vessel function. The responses of pulmonary artery in the presence of curves of increasing concentrations of NA or ACh were recorded in real time. The concentration curve used was as follows: 10^–8^ M, 3 × 10^–8^ M, 10^–7^ M, 3 × 10^–7^ M, 10^–6^ M, 3 × 10^–6^ M, 10^–5^ M, 3 × 10^–5^ M, 10^–4^ M for both NA and ACh. Following the presence of the vasodilator agent ACh or vasoconstrictor agonist NA, the forces that developed in the vascular wall were measured every 2 min. Finally, the wire tension (mN/mm) developed by the blood vessels in the presence of the vasoconstrictor NA was evaluated and the relaxation capacity of the arteries in the presence of the vasodilator ACh was calculated as a percentage (%) of the maximum value of precontraction to NA. PowerLab 4/26 hardware (ADInstruments) was used for data acquisition and LabChart7 (multi-channel chart recorder) software was used for image recording.

### Measurements of serum inflammatory markers

Blood samples were collected on anticoagulant from the retro-orbital venous plexus from all groups of animals and analysed after centrifugation at 2500 ×*g* for 10 min at 4 °C. Transforming growth factor beta 1 (TGF-β1), Endothelin-1 (ET-1), Interleukin-1 beta (IL-1β) and Tumor Necrosis Factor alpha (TNF-α) were evaluated at 4 weeks and at the end of the 3-month experimental period. The tests were performed using commercially available enzyme-linked immunosorbent assay (ELISA) colorimetric kits: Human/Mouse TGF-beta 1 Uncoated ELISA Kit (Invitrogen), ET-1 Kit (R&D Systems), Mouse IL-1β and TNF-α DuoSet ELISA (R&D Systems) with a lower detection limit of 7.8 pg/ml, 0.39 pg/ml, 15.6 pg/ml, respectively 31.3 pg/ml according to the manufacturer's protocol. To determine the optical density of the samples, a microplate reader set to a wavelength at 450 nm with a correction set to 540 nm was used, as suggested by the manufacturer.

### Analysis of different cell types in bronchoalveolar lavage fluid

To isolate bronchoalveolar lavage (BAL) fluid, the hamsters were anesthetized with a solution administered intraperitoneally containing 80 mg ketamine, 10 mg xylazine, 2 mg acepromazine/kg body weight and the neck area was disinfected with 70% ethanol. With the help of a surgical scissors, a longitudinal incision was made in the neck to remove the muscle and salivary glands and to expose the trachea without injuring or cutting other blood vessels, keeping the area clean. With an 18G needle, a small semi-excision was made in the trachea under the laryngeal cartilage between two cartilaginous rings and with a cotton thread, the catheter (consisting of a 20G needle covered by a transparent plastic polypropylene tube) was fixed very well. The insertion of the lavage tube should not be made too low to avoid structural damage to the lung. A syringe was loaded with 1 ml of sterile Hanks′ Balanced Salt Solution (HBSS buffer from Gibco) containing 100 µM EDTA and gently 500 μl of salt/EDTA solution were injected into the lung. While the lavage was being aspirated, a chest massage was performed, and then the syringe was detached from the needle and transferred to a clean collection tube, the process being repeated 8–10 times. A volume of 10 ml salt/EDTA solution was used for each animal. The collected sample was kept on ice. The whole procedure was performed under a stereomicroscope. The tube with the pulmonary aspirate was centrifuged at 800 ×*g* for 10 min at 4 °C. This protocol was adapted after previously described by [30, 31]. The resulting pellet, containing the cellular influx in the lung, was washed with filtered PBS, centrifuged at 800 ×*g* for 10 min at 4 °C and the supernatant was removed. Subsequently, the cell pellet was resuspended in 200 µl Ammonium-Chloride-Potassium (ACK) Lysis Buffer (Gibco) and incubated for 2 min at room temperature (RT), over which 1 ml of PBS was added and again centrifuged. The pelleted cells were resuspended in 500 µl of PBS, counted on the hemocytometer chamber and then labelled with specific antibodies for different cell types: T cells (CD3e^+^, Invitrogen), T helper cells (CD4^+^, Abcam), alveolar macrophages (Singlec-1^+^, SantaCruz Biotechnology), dendritic cells (CD11c^+^, SantaCruz Biotechnology) and proinflammatory macrophages (MHC-II^+^, Invitrogen). After a 40-min incubation at RT in the dark, the cells thus marked were analysed by flow cytometry. The analysis of the different cell types in the BAL fluid was done according to the protocol described by Van Hoecke et al. [32]. For all these markers, the gates were set using the unmarked sample. A total of 10,000 events were counted for each sample.

### Western blot analysis

*Protein extraction:* Snap-frozen pieces of the pulmonary artery were processed for protein extraction. For this procedure, the samples were finely ground with a pair of scissors, then immersed in RIPA buffer (Thermo Scientific) containing 100 mM PMSF and a cocktail of phosphatase B inhibitors and protease inhibitors, and finally they were homogenized using 1 mm diameter glass beads and Minilys Personal High Power Tissue Homogenizer (Bertin Technologies) at 5000 rpm × 10 rounds for 2 min each with a 2 min break on ice. After processing, the samples were kept for 3 h at − 80 °C, then allowed to thaw at RT and centrifuged at 15,600 rpm, for 5 min, at 4 °C. The supernatant containing the cytosolic protein fractions (protein lysate) was collected in new tubes and the protein concentration was quantified using the BCA Protein Assay Kit (ThermoScientific). Sample replicates with a working range of 20–2000 µg/ml were measured at a wavelength of 562 nm on a reading plate and reported on a standard curve of bovine serum albumin (BSA).

*Gel electrophoresis and immunoblotting:* Equal amounts of protein (100 μg/lane) were separated by 8%-12% SDS-PAGE gel electrophoresis under denaturing conditions and transferred onto nitrocellulose membrane. For each set of experiments, a wide range molecular weight marker (6.5—200 kDa) (Sigma-Aldrich) was loaded into one or two lanes serving as standard. Blockade of nitrocellulose membranes was performed prior to antibody labelling in a non-specific site blocking solution (TBST solution with 3% BSA (AppliChem) or TBST solution with 5% Blotto, non-fat dry milk (Santa Cruz Biotechnology)) for 1 h at RT. The membranes were incubated at 4 °C overnight with the following primary monoclonal antibodies: AP-1 28 kDa (1:500, Sigma-Aldrich), pFAK 125 kDa (1:200, SantaCruz Biotechnology), FAK 125 kDa (1:200, SantaCruz Biotechnology), pERK 42/44 kDa (1:1000, R&D Systems), ERK 42/44 kDa (1:200, Abcam), and incubated for 1 h with β-actin 42 kDa or GAPDH 36 kDa—used as loading control (1:200, SantaCruz Biotechnology/ 1:1000, Abcam 37168) which recognize the proteins of interest. The membranes were washed thoroughly three times with TBST (Tris Buffered Saline and 0.05% Tween 20) and subsequently incubated with horseradish peroxidase-conjugated secondary antibodies: anti-mouse antibodies (1:5000, ThermoFisher Scientific) or anti-rabbit antibodies (1:5000, ThermoFisher Scientific), which specifically recognize primary antibodies, for 1 h at RT. Detection was performed with enhanced chemiluminescence reagents (ECL) (AppliChem) and the protein bands were quantified with the TotalLab program. The intensity of the bands was normalized to β-actin/GAPDH levels, a housekeeping reference protein or to the total form of the protein.

### Transmission electron microscopy

Under deep anesthesia, the animals were perfused through the left ventricle with modified Karnovsky's fixative consisting of 2.5% glutaraldehyde and 1.5% paraformaldehyde in 0.1 M sodium cacodylate buffer (pH 7.2). Small fragments of pulmonary artery, lung and right ventricle free wall were harvested and processed for routine Transmission Electron Microscopy (TEM). Briefly, approximately 1 cubic mm tissue blocks were postfixed with 1% osmium tetroxide for 90 min, and stained *en bloc* with 0.5% uranyl acetate for 30 min. Then, the tissue samples were dehydrated in a graded series of ethanol, cleared in propylene oxide, embedded in Epon 812 epoxy resin, and sectioned for TEM. Ultrathin sections (70 nm) were mounted on copper grids, double-stained with uranyl acetate and lead citrate, and examined in a FEI Tecnai G2 Spirit BioTwin TEM (Eindhoven, The Netherlands) at 100 kV. Digital images were recorded with a bottom-mounted FEI Eagle 4 k CCD camera and its TIA software (Eindhoven, The Netherlands).

### RNA Extraction and Real-Time qPCR analysis

The total RNA was extracted from cryopreserved tissue samples, namely the pulmonary artery and right ventricle, using the miReasy Micro Kit (QIAGEN). For one sample, approximately 10 mg of tissue were minced very well with fine scissors on a metal support held in liquid nitrogen, 700 μl lysis buffer (QIAzol Lysis Reagent) were added, kept in the freezer for 10 min and then 1.4 mm zirconium oxide beads (Precellys) were added. The sample was subjected to grinding cycles at 1800 rpm, 6 times for 1 min on ice. Centrifugation was performed at 10,000 ×*g* for 5 min at 4 °C, and the extract was transferred to a new tube where the total RNA was extracted with chloroform and precipitated with isopropanol and processed according to the manufacturer's recommendations. The elution of the RNA was done in a final volume of 16 μl of RNase-free water, and the RNA thus obtained was kept at − 80 °C until examinations. The purity and concentration of the RNA was read by spectrophotometry using NanoDrop 2000c (ThermoFisher). TaqMan MicroRNA Reverse Transcription Kit (Applied Biosystems) was used for reverse transcription of RNA into cDNA synthesis in combination with TaqMan-Gene Expression Master Mix according to the instructions of the manufacturer on a Veriti real-time PCR system (Applied Biosystems). A total of six miRNAs were analysed, each reaction per sample done in triplicate: hsa-miR-21 (ID:0000397), hsa-miR-124 (ID:000446), hsa-miR-204 (ID:000508), hsa-miR-214 (ID:002306), hsa-miR-210 (ID:000512), hsa-miR-145 (ID:002278). U6 small nucleolar RNA snRU6 (ID: 00197) was used to normalize the expression level of miRNA and quantified using the 2^–∆∆Ct^ calculation method. The VIIA7 Software v1.2 (Applied Biosystems) with the automatic quantification cycle (Cq) setting was used to analyse the data.

### Tissue processing and histological examination

After sampling the organs of interest (pulmonary artery, lung, right ventricle and liver), previously washed well with PBS with Ca^2+^, the tissues were prepared for histopathological examination. For the paraffin sections, the samples were fixed in 4% paraformaldehyde for 24 h at RT, dehydrated in successive ethanol baths, embedded in paraffin, and subsequently they were cut with a microtome at a thickness of 5 µm. Before staining, the sections were kept in oven at 60 °C, deparaffinized and rehydrated in decreasing graded xylene and ethanol baths (from 100 to 70%), washed with distilled water and then mounted on SUPERFROST PLUS glass slides (ThermoScientific) treated with Poly-l-Lysine. The paraffin sections were then subjected to Hematoxylin&Eosin (H&E) or Picro-Sirius Red staining (AB 245887 kit, Abcam) to observe the presence of collagen, the main indicator of fibrosis, but also to observe vascular remodelling. Hematoxylin is a basic dye with affinity for acidic components of the cell, Eosin is an acidic dye with affinity for basic cellular components, and Picro-Sirius is one of the most important stains to study collagen networks in different tissues, in particular collagen I and III fibers. All slides were observed and imaged using bright-field microscope (Leica DMi8, software LAS X) at 20 × magnification.

### Immunofluorescence analysis

Immunofluorescent staining was performed on tissue samples (pulmonary artery, lung, right ventricle and liver) in order to monitor inflammatory and fibrotic markers. Immediately after harvesting, they were fixed in cryoprotection solution of 2% paraformaldehyde in 0.1 M phosphate buffer and left at 4 °C overnight. To prepare the histological sections, the tissue was passed through consecutive baths of glycerol of different concentrations (5% for 15 min at RT, 10% for 1 h at 4 °C, 20% overnight at 4 °C and 50% for 1 h at 4 °C), then washed with a solution of 3% sucrose, 6 times for 15 min, and immersed in OCT (Tissue-Tek, Sakura) for 30 min. To be mounted on the cutting support, the samples were quickly frozen in liquid nitrogen and cut with a cryotome (Leica CM1850) using special blades (MX35 Ultra, ThermoScientific), into 5 µm thick sections and attached to special SUPERFROST PLUS glass slides (ThermoScientific) treated with Poly-l-Lysine. The immunostaining process consisted of acclimatization of the sections at RT, fixation in methanol (− 20 °C), quenching of autofluorescence with sodium borohydride for 1 h at 4 °C, permeabilization with 0.2% Triton X-100 (ROTH) in PBS with 0.05% Tween 20 (AppliChem) for 30 min at RT and blocking non-specific sites with 10% goat serum (Invitrogen). The sections were separated on the slide using a lipid pen (Invitrogen) and labeled overnight at 4 °C with the following primary antibodies (diluted in PBS with 1% BSA) against: COL1A (1:250, Santa Cruz Biotechnology), α-SMA (1:200, Cell Signaling Technology), Cx43 (1:200, Thermo Fisher Scientific), MMP-9 (1:200, Santa Cruz Biotechnology), Phalloidin-FITC (5 µg/ml, Sigma-Aldrich), PECAM-1 (CD31) (1:200, Santa Cruz Biotechnology), VE-cadherin (1:200, Santa Cruz Biotechnology), OB-cadherin (1:200, Santa Cruz Biotechnology), CTGF (1:200, Santa Cruz Biotechnology), Fibronectin (1:200, Invitrogen), Vimentin (1:200, Santa Cruz Biotechnology), washed 3 times with PBS, incubated with secondary antibodies, Alexa Fluor 647 donkey anti-mouse IgG (H + L) and Alexa Fluor 488 goat anti-rabbit IgG (H + L) (1:500, Invitrogen) for 1 h, and then washed 3 times with PBS. Nuclei were stained with 4′,6-Diamino-2-phenylindole (DAPI) solution (5 mg/ml in PBS − 10 mM) for 5 min, washed 3 times with PBS and mounted with ProLong solution (Invitrogen) and allowed 24 h to polymerize at RT. To assess ROS (reactive oxygen species) levels, 6 µM of fluorochrome dihydroethidium (DHE) (Sigma-Aldrich) were added on tissue sections for 30 min at RT. All images were captured and analysed under a fluorescence microscope (Axio Vert.A1 Fl, Carl Zeiss, software Axio Vision Rel 483SE64-SP1) at 20X magnification. Image analysis was performed using ImageJ program.

### Data analysis

For the quantification and comparison of the data, One-Way ANOVA, Bonferroni post-test analysis method in GraphPad Prism 8.0 programme was applied. The statistically significant differences between the groups were calculated, and represented as ***P < 0.005, **P < 0.01, *P < 0.05 for values vs. control group, and ^###^P < 0.005, ^##^P < 0.01, ^#^P < 0.05 for values vs. MCT group.

## Results

### The effects of MCT injection and siRNA AP-1 treatment on plasma biochemical parameters

For the characterization of the three experimental groups in terms of biochemical parameters, blood was taken from the retro-orbital venous plexus at 4 and 12 weeks respectively after the subcutaneous injection of MCT (60 mg/kg body weight). Accordingly, the values of blood glucose, cholesterol, HDL-cholesterol, LDL-cholesterol, triglycerides, hepatic transaminases, and the final weight of the animals were analysed (Table 1). The hamsters’ body weight remained relatively constant between the three groups of animals, with a slight tendency to decrease in the MCT group, but without statistical significance. The heart of the animals was also weighed at the end of the 12 experimental weeks, and the results showed in Table 1 revealed a significant increase in the MCT group (***P < 0.005) compared to a considerable decrease following the administration of the siRNA AP-1 treatment (^##^P < 0.01). At 4 weeks, the values of all the biochemical parameters showed no differences between the groups, the values remaining within a normal range (Table 1). At 12 weeks, the MCT injection induced a significant increase (***P < 0.005) in triglyceride values compared to the control group. These values were significantly reduced (^###^P < 0.005) in the case of the group that received siRNA AP-1 treatment. For the rest of the investigated parameters, there was a slight upward trend for the MCT group, but the values did not show statistical significance. The plasma values of hepatic transaminases (AST and ALT expressed as (U/L)) remained within normal limits both at 4 and 12 experimental weeks, so that the subcutaneous injection with MCT did not affect liver function, which means that the selected dose of 60 mg/kg body for MCT injection had no toxic effect on the liver (Table 1).

**Table 1:** Evaluation of biochemical parameters of the three experimental animal groups: Control (C), MCT group, and MCT-siRNA AP-1 group, after 4 respectively 12 weeks of MCT injection to induce pulmonary arterial hypertension

After 4 weeks of MCT injection/2 weeks of siRNA AP-1 treatment	Control group (n = 12)	MCT group (n = 14)	MCT-siRNA AP-1 group (n = 13)
Glycaemia (mg/dl)	150.29 ± 35.4	165.43 ± 28.4	143.78 ± 19.4
Cholesterol (mg/dl)	203.58 ± 30.3	181.74 ± 14.6	162.63 ± 23.3
Triglyceride (mg/dl)	204.22 ± 26.6	340.89 ± 54.5	214.52 ± 25.6
HDL cholesterol (mg/dl)	70.56 ± 11.5	64.73 ± 11.8	66.66 ± 14.9
LDL cholesterol (mg/dl)	81.8 ± 8.65	80.41 ± 9.3	87.71 ± 15.8
Hepatic transaminases (U/L)			
AST	84.72 ± 3.72	89.57 ± 4.8	92.97 ± 4.4
ALT	78.45 ± 1.7	80.21 ± 4.0	84.91 ± 2.6

After 12 weeks of MCT injection/10 weeks of siRNA AP-1 treatment	Control group (n = 12)	MCT group (n = 14)	MCT-siRNA AP-1 group (n = 13)
Final body weight (g)	121.42 ± 11.9	107.66 ± 10.3	110.64 ± 11.8
Heart weight (g)	0,49 ± 0.05	**0.98 ± 0.10** ***P < 0.005	**0.65 ± 0.06** ^**##**^**P < 0.01**
Glycaemia (mg/dl)	136.45 ± 19.9	160.19 ± 28.2	139.92 ± 19.7
Cholesterol (mg/dl)	177.69 ± 14.6	190.70 ± 18.4	164.09 ± 16.1
Triglyceride (mg/dl)	225.23 ± 25.1	**430.83 ± 85.9** ***P < 0.005	**173.45 ± 44.1** ^**###**^**P < 0.005**
HDL cholesterol (mg/dl)	60.37 ± 11.6	71.75 ± 6.1	60.25 ± 7.8
LDL cholesterol (mg/dl)	72.20 ± 17.1	88.83 ± 9.5	77.61 ± 12.3
Hepatic transaminases (U/L)			
AST	100.43 ± 6.6	100.83 ± 5.8	100.18 ± 3.9
ALT	93.94 ± 8.6	95.16 ± 4.9	97.22 ± 4.1

Levels of body weight (g), heart weight (g), plasma glucose, total cholesterol, LDL-cholesterol, HDL-cholesterol, and triglycerides, were all expressed as mg/dl and hepatic transaminases as U/L. Data are means ± SD of duplicate determinations

The statistical significance, noticeably different, was represented as ***P < 0.005 values versus control group and ^###^P < 0.005, ^##^P < 0.01 values versus MCT group. One-way ANOVA, Bonferroni post-test was applied

### The effects of MCT injection and siRNA AP-1 treatment on pulmonary arterial hypertension

**Fig. 2:**
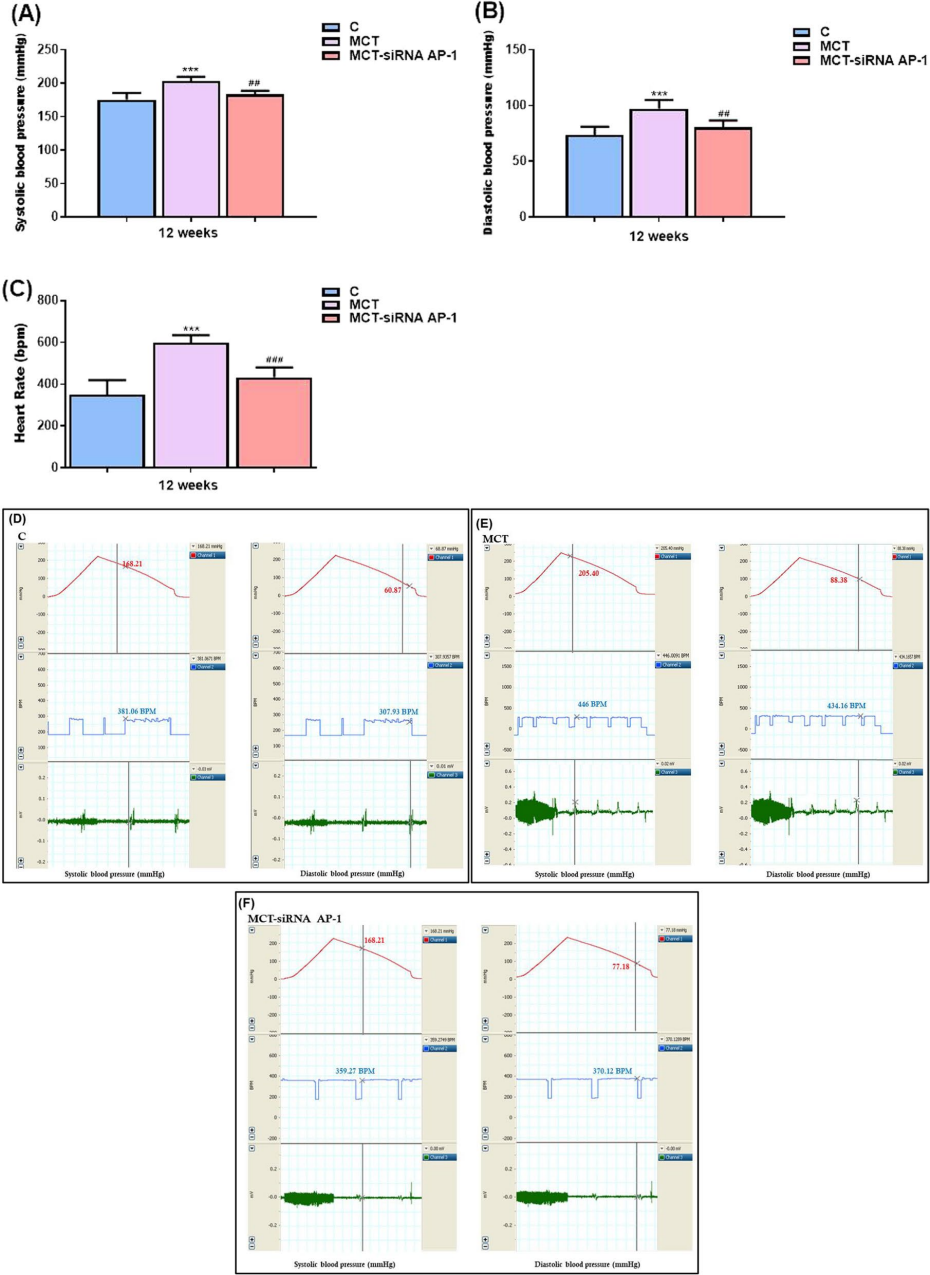
The development of pulmonary arterial hypertension in hamsters exposed to MCT for 12 weeks and siRNA AP-1 for 10 weeks compared to control animals. **A** Systolic blood pressure (mmHg), **B** diastolic blood pressure (mmHg) and **C** Heart Rate (bpm) of pulmonary arteries were measured using non-invasive voltage device and data quantification. For each measurement, the average value of a continuous recording interval (8–10 times) was calculated. Five animals from each experimental group were evaluated. The data represent the means ± SD. Statistical significance represented as ***P < 0.005, versus control group and ^###^P < 0.005, ^##^P < 0.01 versus MCT group. One-way ANOVA, Bonferroni post-test was applied. **D**–**F** Representative waveforms of pulmonary arterial pressure in all three experimental animal groups: systolic blood pressure (left-red), diastolic blood pressure (right-red), heart rate (blue)

It is known that the main effect of MCT is to induce pulmonary arterial hypertension within 2–3 weeks after administration. In our experimental groups of hamsters, systolic and diastolic blood pressure and heart rate were monitored 12 weeks after MCT administration, and 10 weeks after siRNA AP-1 treatment (Fig. 1). In the hamsters in the MCT group, a significant increase in systolic blood pressure was recorded (***P < 0.005) (Fig. 2A, E), which correlated with significant increases in diastolic blood pressure (***P < 0.005) (Fig. 2B, E), and heart rate (***P < 0.005) (Fig. 2C, E). Additionally, it can be seen from the measured data that the siRNA AP-1 treatment group displayed significantly lower blood pressure (systolic and diastolic) (^##^P < 0.01) and heart rate values (^##^P < 0.005) that were relatively close to those of the control group (Figs. 2A,B,C,D,F). The hamsters' heart rates ranged from 381 beats per minute (bpm) in the control group to 446 bpm in the MCT group before dropping back to 359 bpm in the treated group with siRNA AP-1. The heart rate was also assessed and monitored at the time of the echocardiographic analysis.

### Pulmonary artery function in MCT-induced PAH and siRNA AP-1 treatment effects evaluated by wire myography

**Fig. 3:**
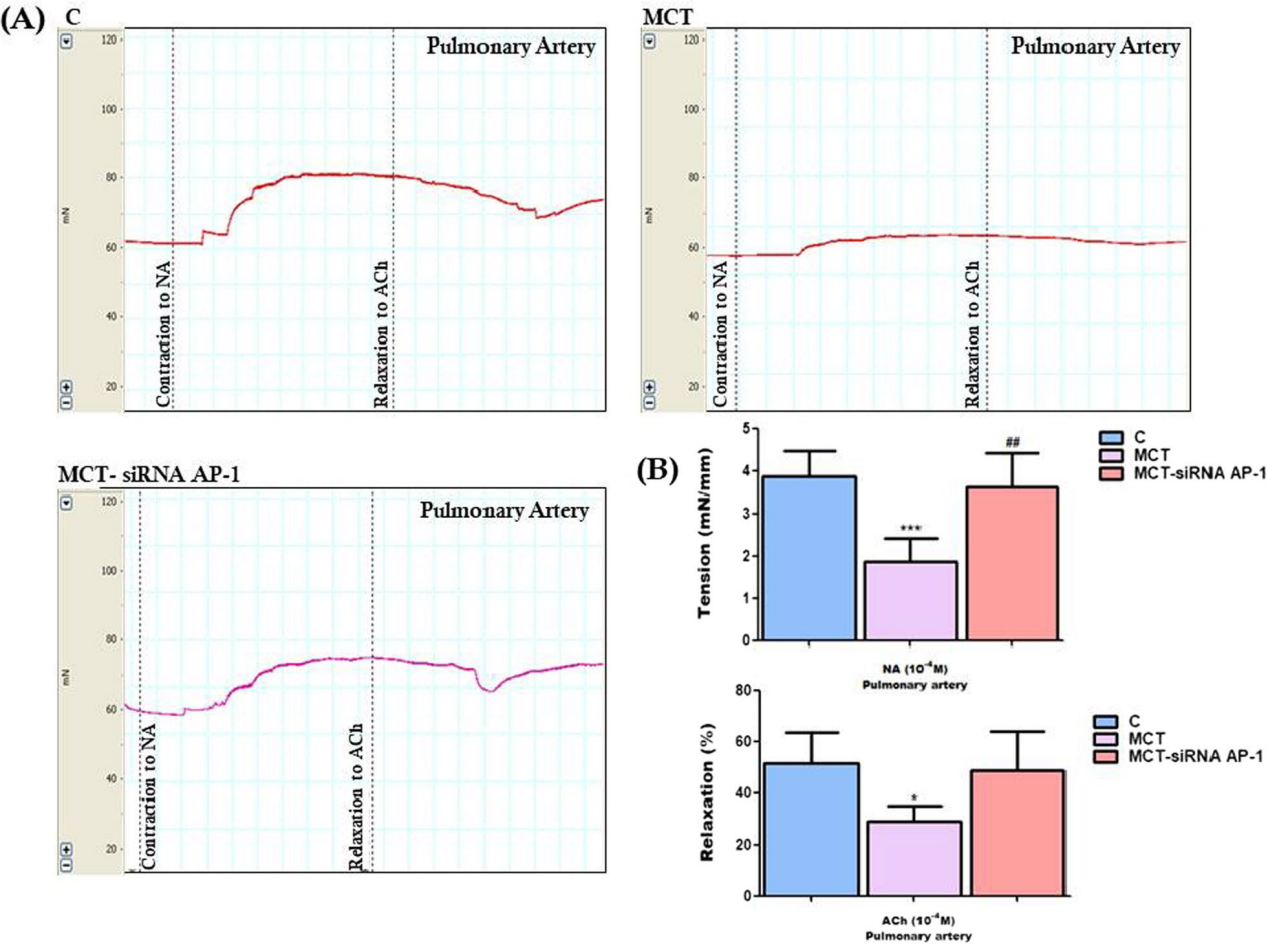
**A** Representative images with myographic recordings at selected time points: for the contraction at NA (10^−8^ M ÷ 10^−4^ M) and relaxation at ACh (10^−8^ M ÷ 10^−4^ M) of the pulmonary arteries isolated from all three investigated experimental groups. Images were recorded with LabChart 7 software. **B** Measures of maximal contraction forces developed by pulmonary arteries to 10^–4^ M NA and maximal relaxation percentages to 10^–4^ M ACh for the three hamster groups. Data are means ± SD of 4 independent experiments for each investigated group. The statistical significance, noticeably different, was represented as ***P < 0.005, *P < 0.05 versus control group and ^##^P < 0.01 versus MCT group. Probability values were calculated by One-way ANOVA, Bonferroni post-test

In order to follow the functionality of the pulmonary arteries, the myograph technique which records in real time the contraction and relaxation capacity of the arterial wall was used. In this way, the effects of both MCT injection and siRNA AP-1 treatment were investigated on endothelial and muscle cells present in the structure of the vascular arterial wall originated from the three hamster groups (C, MCT, MCT-siRNA AP-1) at the end of the 12-weeks experimental period. The contraction and relaxation capacity of smooth muscle cells (SMCs) is responsible for the control and regulation of vascular tone, and any disturbance in their function is attributed to damage of the vascular endothelium. The records obtained with the help of LabChart 7 software for each individual experimental group highlighted that the maximum values both for the contraction induced by NA and the relaxation at ACh were obtained for the concentration of 10^−4^ M (Fig. 3). Regarding the MCT group, the ability of the pulmonary arteries to contract and relax to the action of the vasoconstrictor and vasodilator agents was significantly affected compared to the control group (***P < 0.005, respectively *P < 0.05) (Fig. 3). These functional changes correlate with the onset of vascular endothelial dysfunction established following MCT administration. After treatment with siRNA AP-1 (MCT-siRNA AP-1 group), vascular reactivity of the pulmonary arteries was visibly restored, especially the response to the vasoconstrictor agent, to values close to those recorded in the control animals (^##^P < 0.01). Additionally, the ability of pulmonary arteries to relax was also improved, but without statistical significance (Fig. 3). In our experiments dedicated to investigating the functionality of the pulmonary arteries, NA-induced contraction (10^−8^ M ÷ 10^−4^ M) was measured as the tension developed in the vascular wall (mN/mm), and endothelium-dependent relaxation to ACh (10^−8^ M ÷ 10^−4^ M) was calculated as a percentage of the maximum NA pre-contraction.

### Structure and function of pulmonary artery and right ventricle in MCT-induced PAH and siRNA AP-1 treatment effects monitored by echocardiography

**Fig. 4:**
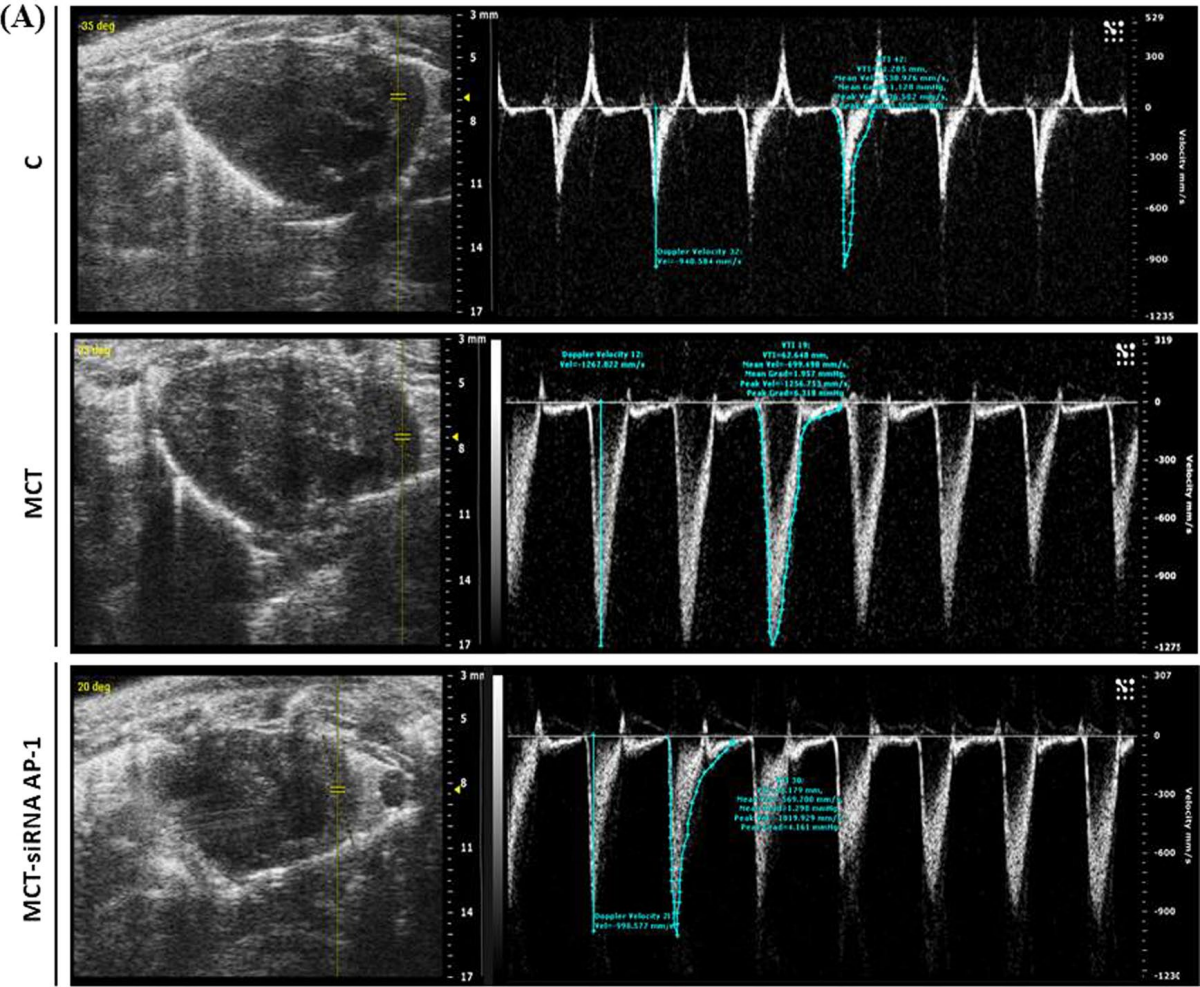

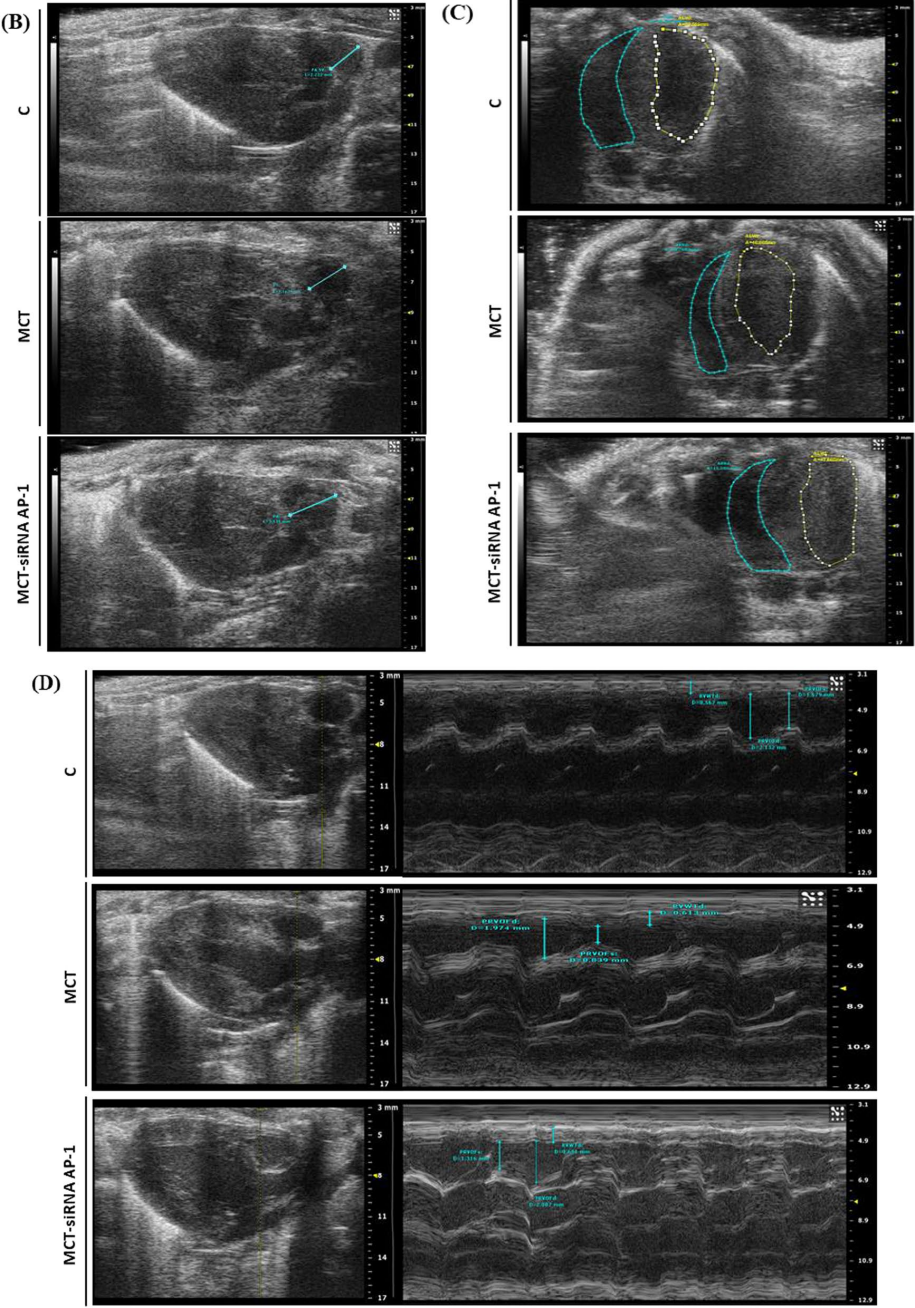

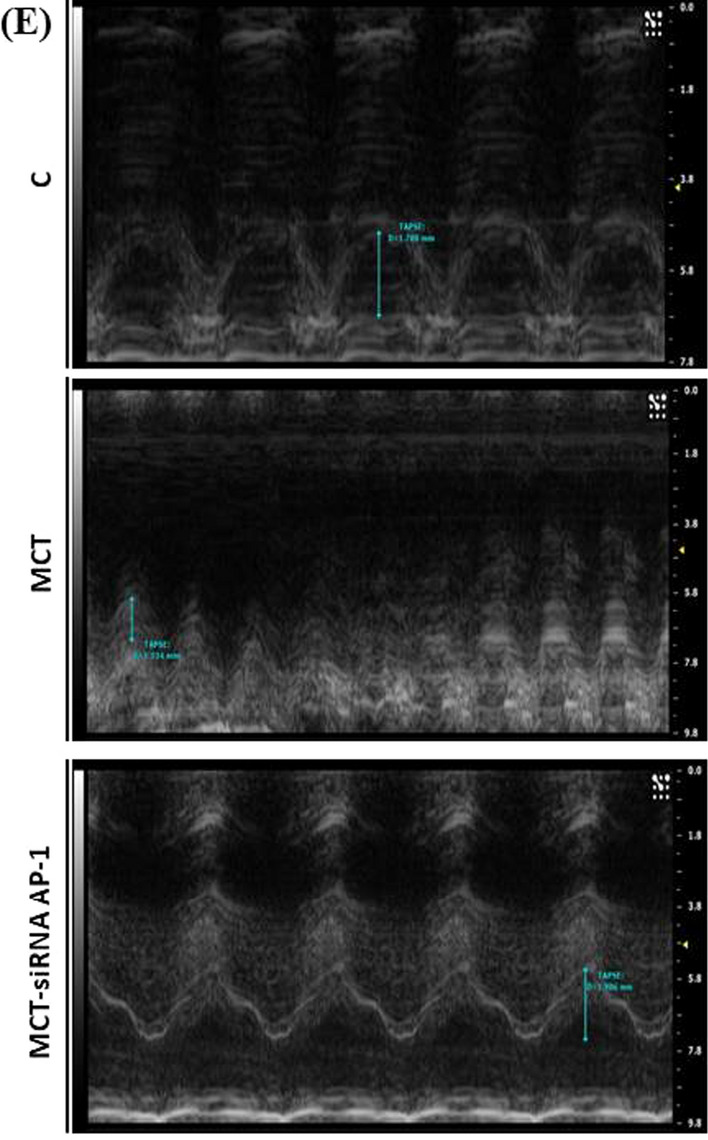
Changes in the blood flow, structure and function of pulmonary artery and right ventricle for the investigated experimental groups: C, MCT, MCT-siRNA AP-1. **A** Representative recordings obtained in pulsed wave Doppler-mode, which highlight the Velocity Time Integral (VTI, mm) and Doppler Peak Velocity (Vel, mm/s) of the pulmonary artery; **B** representative B-mode recordings, which highlight the inner diameter (mm) of the pulmonary artery; **C** Representative B-mode recordings, which highlight the area of the right and left ventricle in diastole (mm^2^); **D** Representative M-mode recordings, which highlight right ventricle wall thickness in diastole (RVWTd, mm); Representative M-mode recordings, which highlight proximal ventricle outflow chamber during systole and diastole (PRVOFd, PRVOFs, mm); **E** Representative M-mode recordings which highlight tricuspid annular plane systolic excursion (TAPSE, mm)

Further in our experiments the echocardiography was used to investigate the changes in the structure and function of the pulmonary artery and right ventricle generated by subcutaneous injection of MCT at 12 weeks from the administration as well as the treatment with siRNA AP-1 managed for 10 weeks. First, we investigated the hemodynamic of blood flow in pulsed wave Doppler-mode (PW Doppler-mode) using two parameters: Velocity Time Integral (VTI) which represents the length of the ejection tract measured in mm and Doppler Peak Velocity (Vel) which represents the blood flow rate measured in mm/s at the pulmonary artery level (Fig. 4A). Pulsed wave velocity is the most frequently used approach for measuring the pressure speed of the pulse from the heart through the arteries. Data obtained in the PW Doppler-mode (Table 2) showed that the animals injected with MCT had considerably higher values for velocity (***P < 0.005), that can be correlated with an increase in pulmonary arterial pressure reported in our experiments. At the same time, a significant decrease (^#^P < 0.05) was observed for this parameter after AP-1 siRNA administration. For the VTI parameter, the values remain constant for the three experimental groups, but with an increasing tendency for the treated animals (Table 2).

**Table 2:** Echocardiographic assessments for the three hamster groups: C, MCT, MCT-siRNA AP-1

Echocardiographic parameters	C group (n = 5)	MCT group (n = 5)	MCT-siRNA AP-1 group (n = 5)
VTI (mm)	44.21 ± 3.4	48.95 ± 7.90	51.22 ± 9.47
Vel (mm/s)	− 1045.61 ± 40.19	**− 1242.99 ± 28.56** ***P < 0.005	**− 1136.44 ± 35.28** ^**#**^**P < 0.05**
PA diameter (mm)	2.49 ± 0.20	**3.35 ± 0.22** ***P < 0.005	3.14 ± 0.13
ARVd (mm^2^)	15.13 ± 4.39	**12.25 ± 2.42** *P < 0.05	14.83 ± 2.57
ALVd (mm^2^)	49.18 ± 1.55	**40.99 ± 2.75** ***P < 0.005	**47.97 ± 1.2** ^**###**^**P < 0.005**
RVWTd (mm)	0.57 ± 0.06	0.64 ± 0.03	0.63 ± 0.04
PRVOFd (mm)	1.91 ± 0.45	1.93 ± 0.29	2.10 ± 0.20
PRVOFs (mm)	1.56 ± 0.39	**1.09 ± 0.31** **P < 0.01	**1.53 ± 0.21** ^**##**^**P < 0.01**
TAPSE (mm)	1.66 ± 0.18	**1.25 ± 0.18** *P < 0.05	**1.72 ± 0.28** ^**##**^**P < 0.01**

Velocity Time Integral (VTI, mm); Doppler Peak Velocity (Vel, mm/s); PA diameter (mm); area of the right and left ventricle in diastole (ARVd/ALVd, mm2); right ventricle wall thickness in diastole (RVWTd, mm); proximal ventricle outflow chamber during systole and diastole (PRVOFd, PRVOFs, mm); tricuspid annular plane systolic excursion (TAPSE, mm). Data are shown as the mean ± SD of each experimental group. Five animals from each group were followed

The statistical significance, noticeably different, was represented as ***P < 0.005, **P < 0.01, *P < 0.05 versus control group and ^###^P < 0.005, ^##^P < 0.01, ^#^P < 0.05 versus HH group. The values were calculated by Owo-way ANOVA, Bonferroni post-test

The structure of the pulmonary artery was investigated using B-mode recordings (Fig. 4B). The data reported in Table 2 revealed that the inner diameter of the pulmonary artery measured in mm showed a significant thickening (***P < 0.005) in the group injected with MCT compared to the control animals, results that correlate with a progressive remodelling accompanied by perivascular fibrosis leading to arterial stiffening. The artery wall structure did not improve as a result of the AP-1 siRNA treatment (Table 2). According to parasternal short-axis views, we measured the structure of the right ventricle performed in B- mode (Fig. 4C) and the data presented in Table 2 revealed that the area of the right ventricle in diastole measured in mm^2^ decreased significantly in the MCT-injected group (*P < 0.05), which can be correlated with an installed right ventricular hypertrophy after the treatment, but also a slight increasing trend in the MCT-siRNA AP-1 group, but without statistical significance. At the level of the left ventricle, a significant decrease in the diameter of the internal cavity in diastole could be observed in the group treated with MCT (***P < 0.005), together with a noticeable increase in siRNA AP-1-treated group (^###^P < 0.005) (Table 2).

The relative thickness of the right ventricular wall, measured in mm, was evaluated in M-mode (Fig. 4D), and from the recordings acquired, an increasing tendency could be observed in the MCT group, but without statistical significance. Each of the three experimental groups provided comparable results. With the help of M-mode, we examined another parameter (PRVOF) which indicates the proximal opening capacity of the right ventricle in both systole and diastole, recordings showed in Fig. 4D. The values measured in diastole were kept constant for all three experimental groups. According to the data presented in Table 2, the values of this parameter measured in systole decreased significantly (**P < 0.01) in the case of animals treated with MCT, but this function was improved after treatment with siRNA AP-1, group that exhibited significantly elevated values (^##^P < 0.01). Through the recordings in M-mode (Fig. 4E) we evaluated the function of the tricuspid valve through the parameter called TAPSE, which provides information on the global function of the right ventricle. This parameter measures the extent of the systolic movement of the lateral portion of the tricuspid ring towards the apex and it is known that in pathology its values decrease. In the case of our measurements presented in Table 2, a statistically significant decrease (*P < 0.05) could be observed in the MCT-treated animals and a significant improvement (^##^P < 0.01) could be seen in the group that received siRNA AP-1 treatment.

### The MCT-induced ultrastructural changes in pulmonary artery, lung and right ventricle were reduced by the siRNA AP-1 treatment

**Fig. 5:**
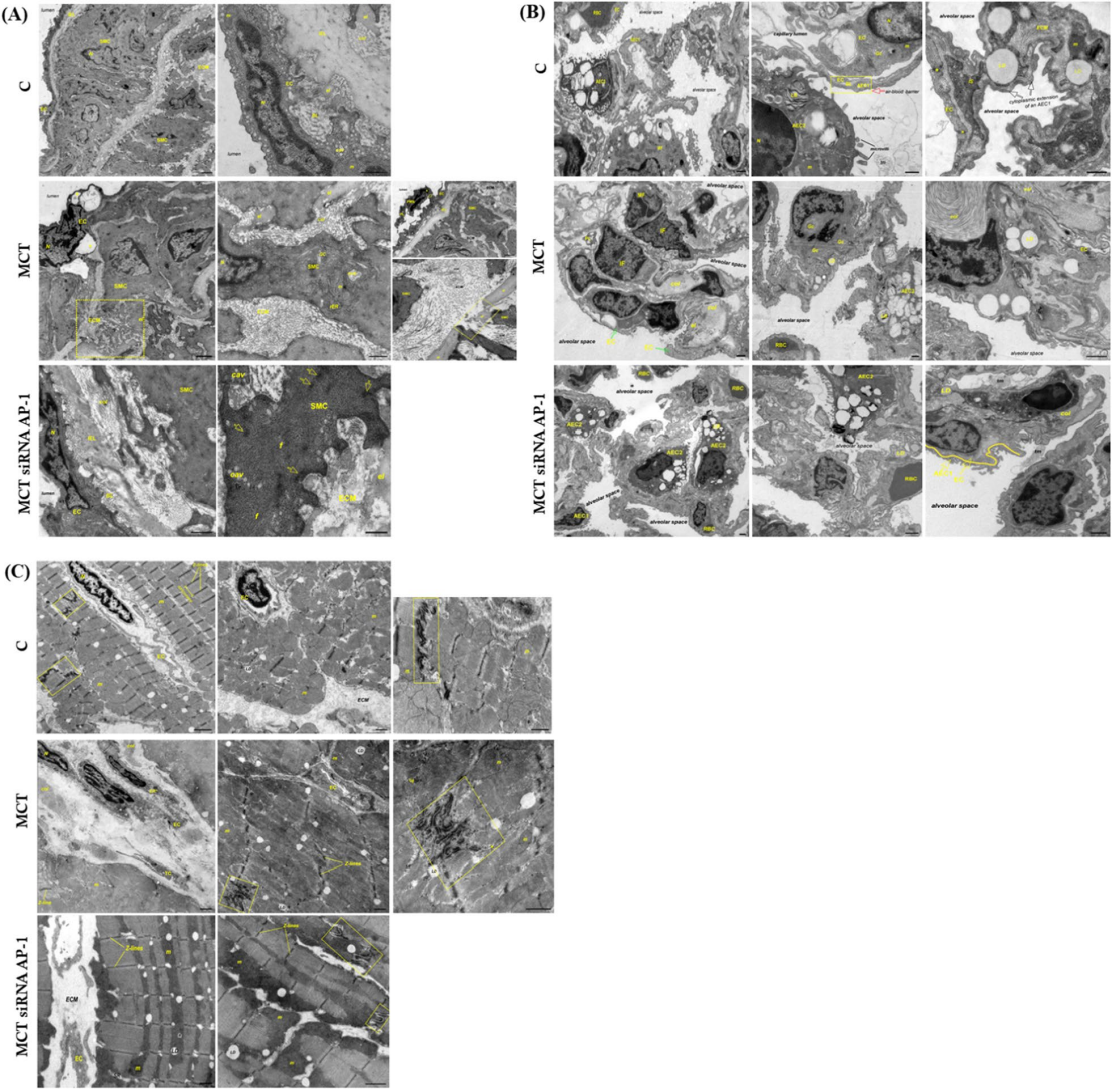
Representative transmission electron microscopy (TEM) images showing the effect of the 12-week administration of MCT alone or in combination with siRNA AP-1 (for 10 weeks) on: **A** the pulmonary artery; **B** lung parenchyma; **C** right ventricle of hamsters from each of the three experimental groups. *EC* endothelial cell, *SMC* vascular smooth muscle cell, *IEL* internal elastic lamina, *BL* basal lamina, *ECM* extracellular matrix, *col*, collagen fibres, *el* elastin, *N* nucleus, *m* mitochondria, *cav* caveolae, *Gc* Golgi complex, *rER* rough endoplasmic reticulum (ER*: stressed ER), *mvb* multivesicular bodies, *PMN* polymorphonuclear leukocyte, *PL* platelet, *f* myofilaments, *AEC 1* type 1 alveolar epithelial cell, *AEC 2* type 2 alveolar epithelial cell, *RBC* red blood cell, *MF* macrophage, *IF* inflammatory cell, *IS* interstitial space, *IC* interstitial cell, *LD* lipid droplet, *LB* surfactant-storing lamellar bodies, *tm* tubular myelin, *Gg* glycogen granules, *TC* telocyte, *SR* sarcoplasmic reticulum, #: large vacuoles; yellow ellipse: contact between a leukocyte and the endothelial cell surface; dashed yellow boxes: regions with fragmented elastin; arrows: dense plaques; yellow freehand line: alveolar-capillary fused basement membrane; yellow boxes: intercalated discs. Scale bars indicate from left to right and top to bottom: **A** 2 µm, 0.5 µm; 1 µm, 0.5 µm, 1 µm / 0.5 µm; 1 µm, 0.25 µm; **B** 1 µm, 1 µm, 0.5 µm; 1 µm, 1 µm, 1 µm; 1 µm, 1 µm, 1 µm; **C** 2 µm, 1 µm, 1 µm; 1 µm, 1 µm, 1 µm; 1 µm, 1 µm, 1 µm

In order to gain deeper insight into the structural changes brought by MCT administered alone or in combination with siRNA AP-1, we performed transmission electron microscopy (TEM) on pulmonary artery, lung and right ventricle samples from hamsters of each study group. The TEM analysis indicated a quite normal structure of the pulmonary arteries from control hamsters, with the intima layer containing flat, elongated endothelial cells, the media having mainly contractile (differentiated) SMCs and elastic fibres, and the adventitia comprising typically fibroblasts and connective tissue (Fig. 5A, upper panel). In contrast, all three layers of the pulmonary arteries from MCT-injected hamsters presented increased thickness, a salient feature of hypertension-triggered vascular remodelling. Specifically, many endothelial cells displayed large vacuoles or biosynthetic and pro-adhesive phenotype, the subendothelial space was widened by cells that conceivably migrated, proliferated or transdifferentiated there, the elastic laminae were fragmented, the media comprised hypertrophic “synthetic” (dedifferentiated) SMCs surrounded by an excessively deposited extracellular matrix (ECM), and the adventitia was filled with collagen-forming cells embedded in a collagen-rich ECM (Fig. 5A, middle panel). However, these abnormalities were attenuated following the treatment with siRNA AP-1. Thus, in the group of MCT and siRNA AP-1 co-treated hamsters, the endothelium showed flattened appearance and no pro-adhesive and vacuolated traits, the elastic lamellae were less fragmented, the thickness of media and adventitia layers was clearly reduced mostly on account of a lesser amount of cells and elastic fibres, although an excessive ECM accumulation was still noticed (Fig. 5A, lower panel). As regards the lung tissue, TEM investigation evidenced that control hamsters exhibited regular air-blood barrier, inter-alveolar septa with apparently normal alveolar epithelial type I (AEC1) and type II (AEC2) cells, and moderate amounts of collagen fibrils in the interstitial space; the AEC2 were easily recognized by their characteristic surfactant storing multilamellar bodies and apical microvilli facing the alveolar space (Fig. 5B, upper panel). Conversely, lung samples from the MCT-injected hamsters showed abnormal inter-alveolar septa, with areas of septal thickening and AEC2 with shortened/absent microvilli and almost empty lamellar bodies, suggesting a defective production and/or release of surfactant in the alveolar space. The interstitial space was markedly enlarged because of a build-up of interstitial cells and extracellular matrix components, particularly collagen fibrils and elastin (Fig. 5B, middle panel). Following the siRNA AP-1 treatment, the lung interstitial remodelling noted in the MCT-injected hamsters was minimized. As compared to the MCT group, the MCT-injected hamsters that received siRNA AP-1 presented less collagen and elastin fibres at the level of the alveolar septa and interstitial space, much more AEC2 that retained their microvilli and lamellar bodies, thinner alveolar-capillary membrane, and fewer interstitial cells (Fig. 5B, lower panel). The ultrastructural findings regarding the cardiac tissue are illustrated in Fig. 5C. The myocardial cells from the right ventricular tissue of the control group displayed the characteristic cross-striated pattern given by the lateral registration of the myofibrillar sarcomeres, the repetitive units of myofibrils delimited by Z-lines; the tightly bundled myofibrils were separated by clusters of mitochondria. Adjacent cardiomyocytes were joined by stair-like structures called intercalated disks, acknowledged as highly organized junctional complexes that intermediate the synchronized contraction of the myocardium (Fig. 5C, upper panel). On the contrary, the right ventricular cardiomyocytes from the MCT-treated hamsters exhibited abnormal or loss of sarcomeric registration, with areas having wavy or misaligned Z-lines, wavy or disorganized myofibrils, and disarranged intercalated discs. Noticeable, an excessive deposition of collagen and other ECM components within the myocardial interstitium and around intramyocardial vessels was another deleterious feature found in the MCT group, that suggested a severely affected tissue functionality (Fig. 5C, middle panel). Most of the structural abnormalities seen in the myocardial cytoarchitecture in the MCT-injected hamsters were reversed by the siRNA AP-1 treatment. The right ventricular cardiomyocytes from the MCT- and siRNA AP-1-co-treated hamsters presented the typical organization of myofibrils into registry, and the regular assembly of myofibrils alternating with intermyofibrillar mitochondria. Also, the morphology of the intercalated discs was broadly retained, and the extent of the ECM accumulation was clearly reduced (Fig. 5C, lower panel).

### The histological changes following MCT administration were remedied after siRNA AP-1 therapy

**Fig. 6:**
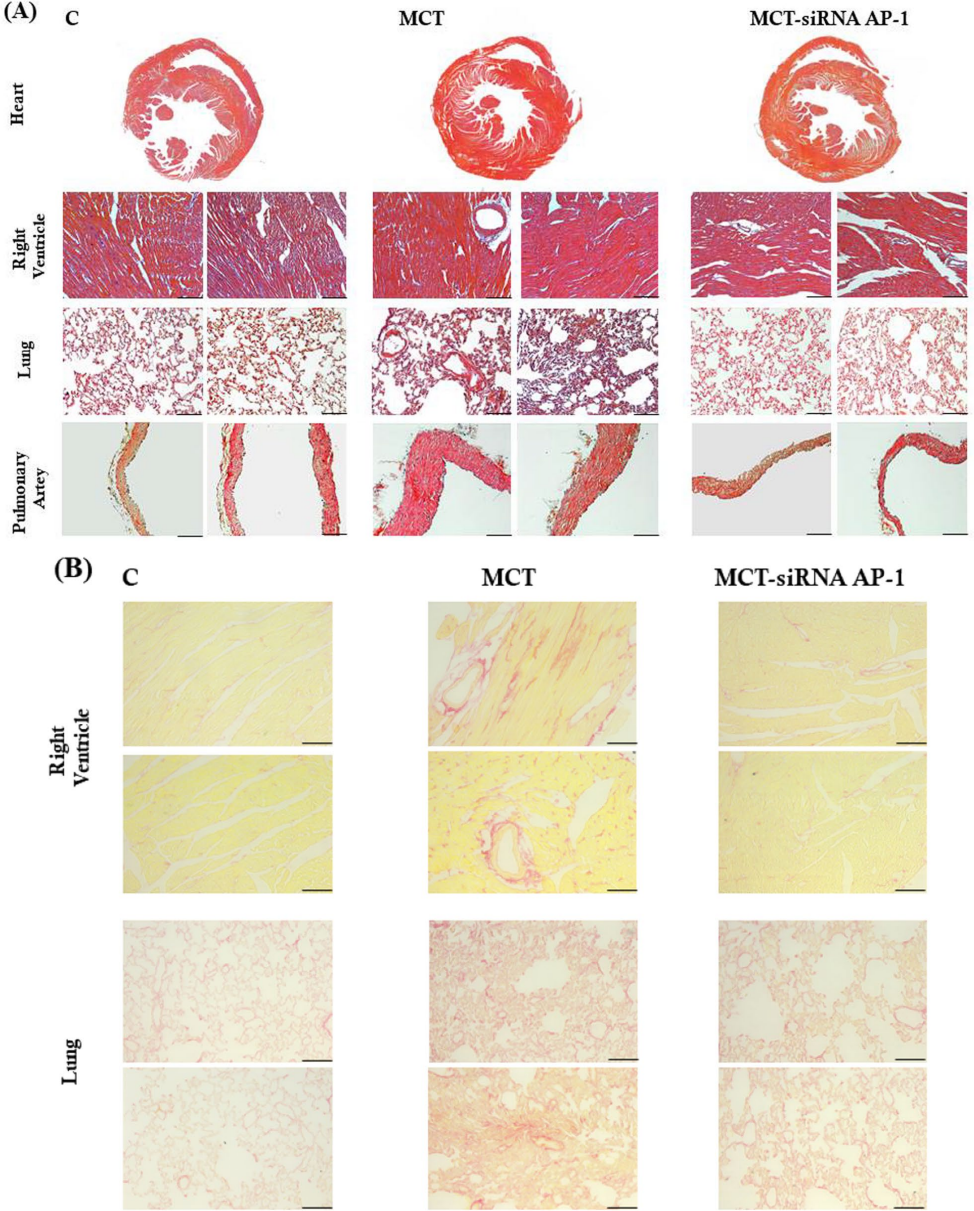
**A** Representative bright-field microscopy images depicting from top to bottom H&E-stained sections with whole heart, right ventricle, lung and pulmonary artery taken from healthy hamsters (C group) and hamsters after 12 weeks of MCT in the absence or presence of siRNA AP-1 injections given for 10 weeks (MCT group and MCT-siRNA AP-1 group). Four different microscopic fields for each experimental point were analysed. Nuclei: blue, cytoplasm: pink, muscle fibres: deep red. **B** Representative bright-field microscopy images depicting Picro- Sirius Red-stained sections with right ventricle and lung taken from healthy hamsters (C group) and hamsters after 12 weeks of MCT in the absence or presence of siRNA AP-1 injections given for 10 weeks (MCT group and MCT-siRNA AP-1 group). Four different microscopic fields for each experimental point were analysed. Nuclei, cytoplasm and muscular fibers are visualized in yellow. Collagen fibers are manifested in a striking manner as vivid orange-red bands. Scale bar indicate: 100 µm, magnification × 20

The structural alterations present in cardiac, lung, and pulmonary vascular tissue were examined on histological sections stained with H&E and Picro-Sirius Red. As a result, H&E images taken on sections of the right ventricle highlighted that in the MCT group, cardiomyocytes displayed an increase in size, a myoarchitectural disarray, a disorganization of the myofibrils with loss of striation, and the wall of the blood vessels appeared thickened in comparison to the normal architecture of the heart tissue from the healthy animals (Fig. 6A). All of these changes are linked to right ventricular hypertrophy and interstitial fibrosis, both improved after siRNA AP-1 treatment. Regarding the lung tissue, there was a thickening of the alveolar wall and alveolar capillary membranes in the group receiving MCT. Also, pulmonary arteries and veins intense congested with obstructions, impaired alveoli, hyperplasic lung parenchyma, and thickened alveolar septa associated with interstitial pulmonary fibrosis were observed in the MCT group. In addition, the structure of pulmonary arterial wall was seriously affected by observing a thickened vascular media, a disordered adventitia, and neointimal proliferation (Fig. 6A). A notable improvement of the mentioned changes in lung and pulmonary vascular tissue was seen in the tissue sections from animals treated with siRNA AP-1, indicating that this therapy restores the cellular and tissue alterations produced following the administration of MCT (Fig. 6A).

Picro-Sirius Red-stained right ventricle and lung tissue structures showed that MCT's influence on these two organs resulted in a notable accumulation of Picro-Sirius Red-positive collagen, myocyte hypertrophy in the right ventricle along with perivascular accumulation of collagen. Thickening of the alveolar wall and alveolar capillary membranes resulting in pulmonary interstitial fibrosis can also be seen on lung sections. In contrast, animals treated with siRNA AP-1 exhibited a significant decrease in foci of Picro-Sirius Red -positive material. All of these changes are compared to tissue samples collected from healthy animals (Fig. 6B).

### Elevated levels of plasma inflammatory cytokines in MCT-induced PAH were alleviated when administering the siRNA AP-1 treatment

**Fig. 7:**
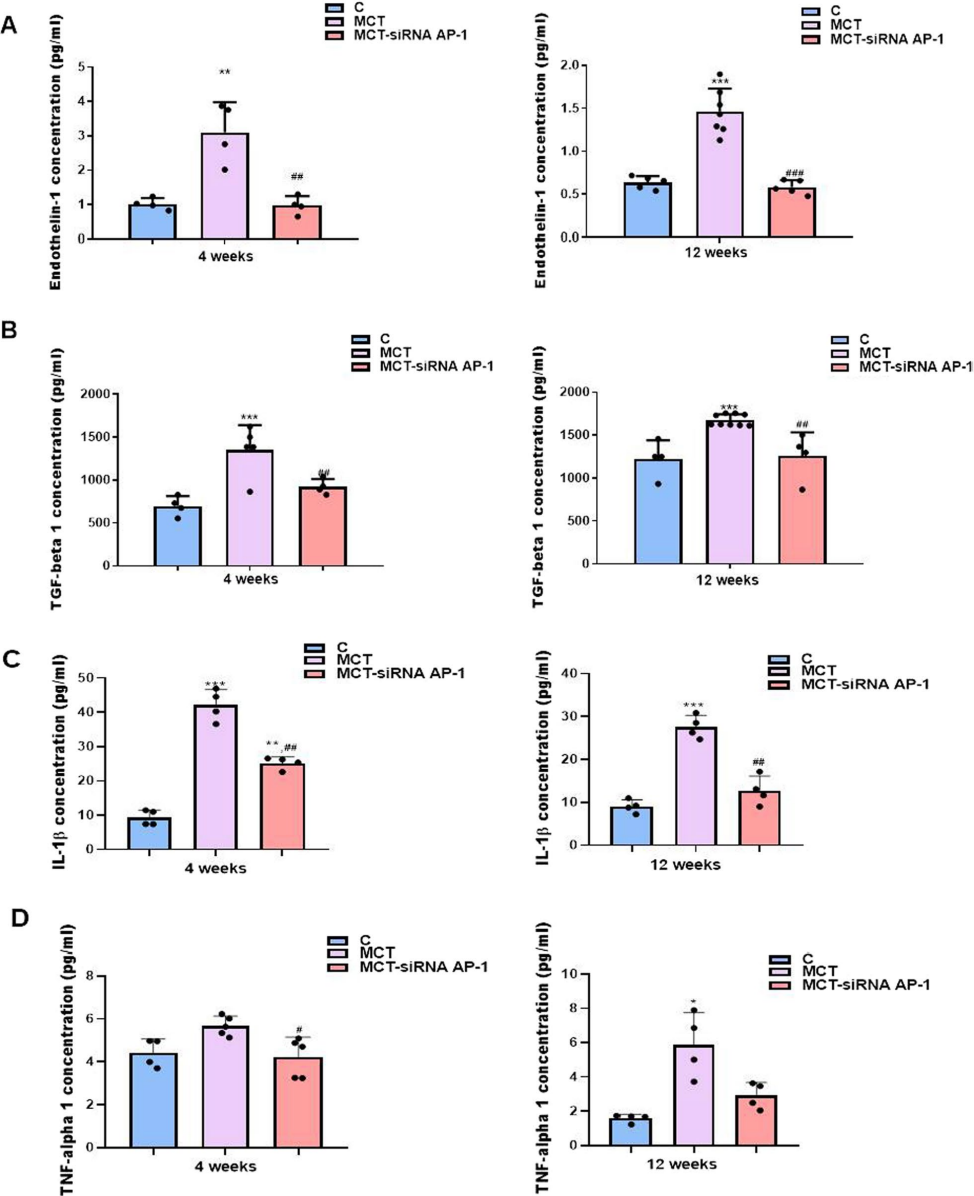
Analysis of plasma inflammatory cytokines at 4 and 12 experimental weeks: **A** ET-1, **B** TGF-β1, **C** IL-1β and **D** TNF-α levels evaluated by enzyme-linked immunosorbent assay (ELISA) method, for all three experimental groups: C, MCT, MCT-siRNA AP-1. The measurements were performed in triplicate and results were depicted as mean ± SD. The statistical significance, noticeably different, was represented as ***P < 0.005, *P < 0.05 versus control group and ^###^P < 0.005, ^##^P < 0.01, ^#^P < 0.05 versus MCT group. The values were calculated by One-way ANOVA, Bonferroni post-test

It is widely established that MCT administration causes an aggravation of inflammatory processes in the vascular and pulmonary systems. In addition, knowing that increased amounts of inflammatory molecules eventually activate the AP-1 transcription complex which is responsible for boosting the proinflammatory and profibrotic factors in cardiopulmonary disease, AP-1 was chosen as primary therapeutic target in MCT-induced PAH. As a result, we selected to investigate the expression of a series of proinflammatory cytokines (ET-1, TGF-β1, IL-1β, TNF-α) in vivo, measured in plasma previously collected from the retro-orbital plexus from all three experimental groups, using ELISA assay (Fig. 7).

The results showed that plasma levels of ET-1 and TGF-β1 are significantly increased both 4 weeks (***P < 0.005) and 12 weeks (***P < 0.005) after MCT administration, and can be correlated with the onset of the changes that have place at the vascular level. A substantial decrease in plasma levels of ET-1 and TGF-1 was detected in the group treated with siRNA AP-1 (^###^P < 0.005; ^##^P < 0.01) (Fig. 7A, B). Similar results were found following the analysis of plasma concentrations of IL-1β and TNF-α (Fig. 7C, D). At 4 experimental weeks of MCT there was a significant increase in plasma IL-1β expression (***P < 0.005), which was maintained at 12 weeks. Throughout the treatment period with siRNA AP-1, the levels of IL-1β remained significantly lower (^##^P < 0.01) with values close to those of the control. In the case of plasma TNF-α expression, an increasing trend was observed at 4 weeks in the MCT group, although statistical significance was apparent after 12 experimental weeks of MCT (*P < 0.05). The siRNA AP-1 treatment started to reduce the expression of TNF-α at 4 weeks (^#^P < 0.05) with a consistent declining trend at 12 weeks (Fig. 7C, D), that correlated with other improvements observed in our experiments.

### Modification of immune and inflammatory infiltrate in bronchoalveolar fluid after MCT and siRNA AP-1 administration

**Fig. 8:**
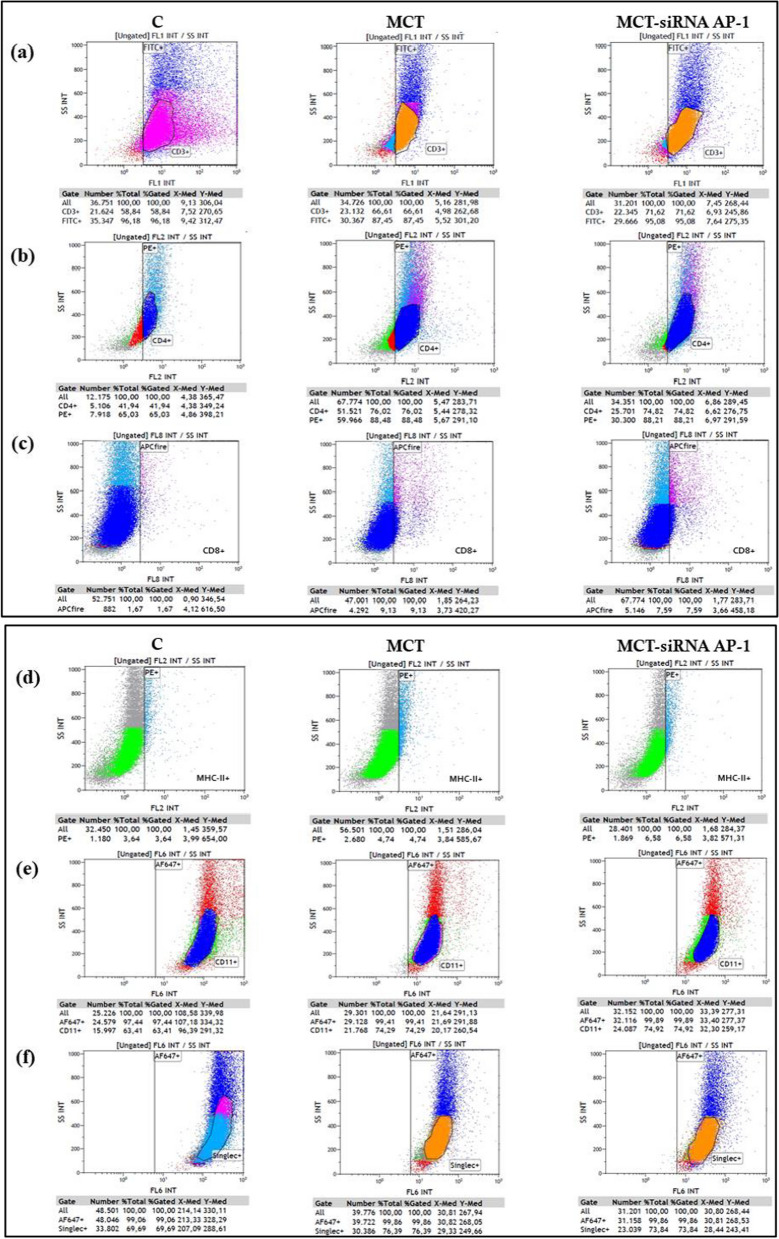
Representative 2-dimensional dot plot graphics of the flow cytometry data for the immune and inflammatory infiltrate in BAL fluid collected from the three hamster groups (C, MCT and MCT-siRNA AP-1). Immune and inflammatory cells were incubated with different antibodies for markers specific to these cell types as follow: **a** CD3e-FITC for T cells, **b** CD4-PE for T helper cells, **c** CD8-APC for T cytotoxic cells, **d** MHC-II-PE for proinflammatory macrophages type M1, **e** CD11c-AF647 for dendritic cells, macrophages, neutrophils, **f** Singlec-1-AF647 for alveolar macrophages. Unstained cells were distributed in the left quadrant and stained cells were distributed in the right quadrant (measurements include the percentage of cells within the gate). A total of 10.000 events counted for each marker

To better understand the immunological status and inflammatory response occurring in the pulmonary airways in cardiopulmonary disease, the immune and inflammatory cell composition of bronchoalveolar lavage (BAL) fluid was examined following administration of MCT alone or in combination with AP-1 siRNA. Therefore, in the cell population isolated from BAL fluid, the expression of a set of markers, such as CD3e (for T cells), CD4 (for T helper cells), CD8 (for cytotoxic T cells), CD11c (for dendritic cells), MHC-II (for proinflammatory macrophages) and Singlec-1 (for alveolar macrophages), was followed by flow cytometry (Fig. 8 (a-f)). Following administration of MCT, the results presented in Table 3 indicate a tendency for an increase in all these investigated markers, with a preponderance for CD4 and CD8 positive immune cell populations that demonstrate a significant increase in comparison to the control group (***P < 0.005). Regarding the outcome of AP-1 siRNA treatment, it had little effect on MCT-induced inflammation, significant decreases being recorded only in the case of CD3 and CD4 positive cell populations (**P < 0.01) (Table 3).

**Table 3:** Analysis of markers specific to inflammatory and immune cells from bronchoalveolar lavage (BAL) fluid by flow cytometry

Markers	C group (n = 5)	MCT group (n = 5)	MCT-siRNA AP-1 group (n = 5)
MHC-II	2.68 ± 1.28	4.03 ± 0.81	**5.96 ± 1.89** **P < 0.01
CD11c	66.16 ± 2.89	75.63 ± 5.98	75.96 ± 3.05
CD3e	55.88 ± 3.80	64.47 ± 10.69	73.54 ± 2.38
Singlec-1	63.63 ± 5.43	76.10 ± 9.09	75.93 ± 1.91
CD4	54.84 ± 18.19	**75.99 ± 4.88** ***P < 0.005	**73.74 ± 2.42** **P < 0.01
CD8	1.69 ± 1.34	**9.98 ± 4.24** ***P < 0.005	**7.05 ± 1.14** **P < 0.01

Five individual experiments were made for each experimental group. The data represent the means ± SD

Statistical significance represented as ***P < 0.005, ^**^P < 0.01 values versus control group. One-way ANOVA, Bonferroni post-test was applied

### Expression level of oxidative stress, remodelling pathway and fibrosis markers was reduced in pulmonary artery, lung, and right ventricle following treatment with siRNA AP-1

In our animal model with arterial hypertension induced by MCT, a variety of ultrastructural, structural and functional changes/abnormalities of the main affected organs, pulmonary artery, lung and right ventricle, was observed. These findings were positively correlated with an increase in the plasma profile of several proinflammatory cytokines that promote inflammation in these tissues. Knowing that tissue remodelling and fibrosis are associated with inflammation, certain specific molecules involved in vascular remodelling, right ventricular hypertrophy, pulmonary and cardiac fibrosis associated with the epithelial-mesenchymal transition (EMT) or fibroblast-to-myofibroblast transition (FMT) were evaluated after MCT administration alone or in combination with AP-1 siRNA treatment.

**Fig. 9:**
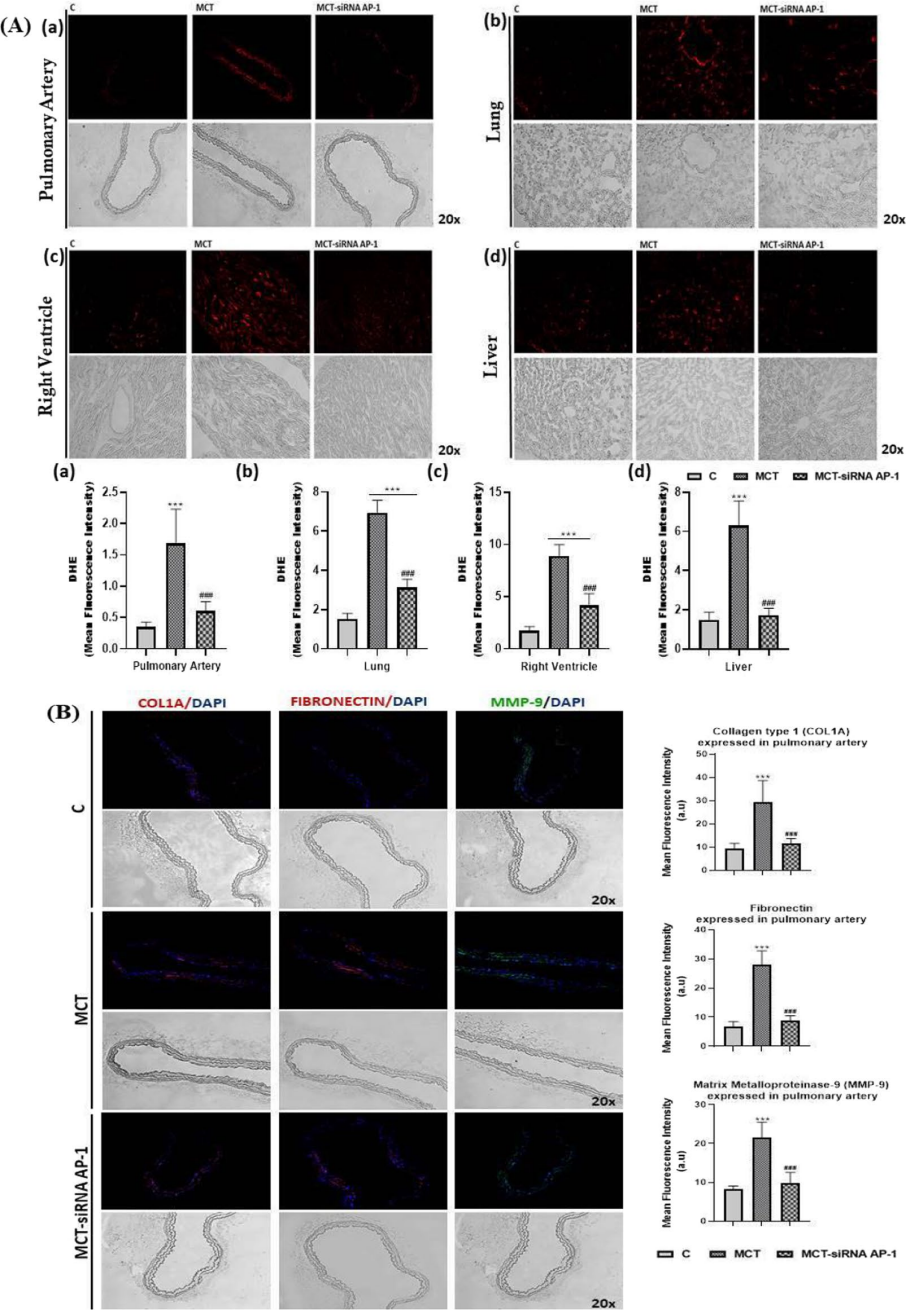

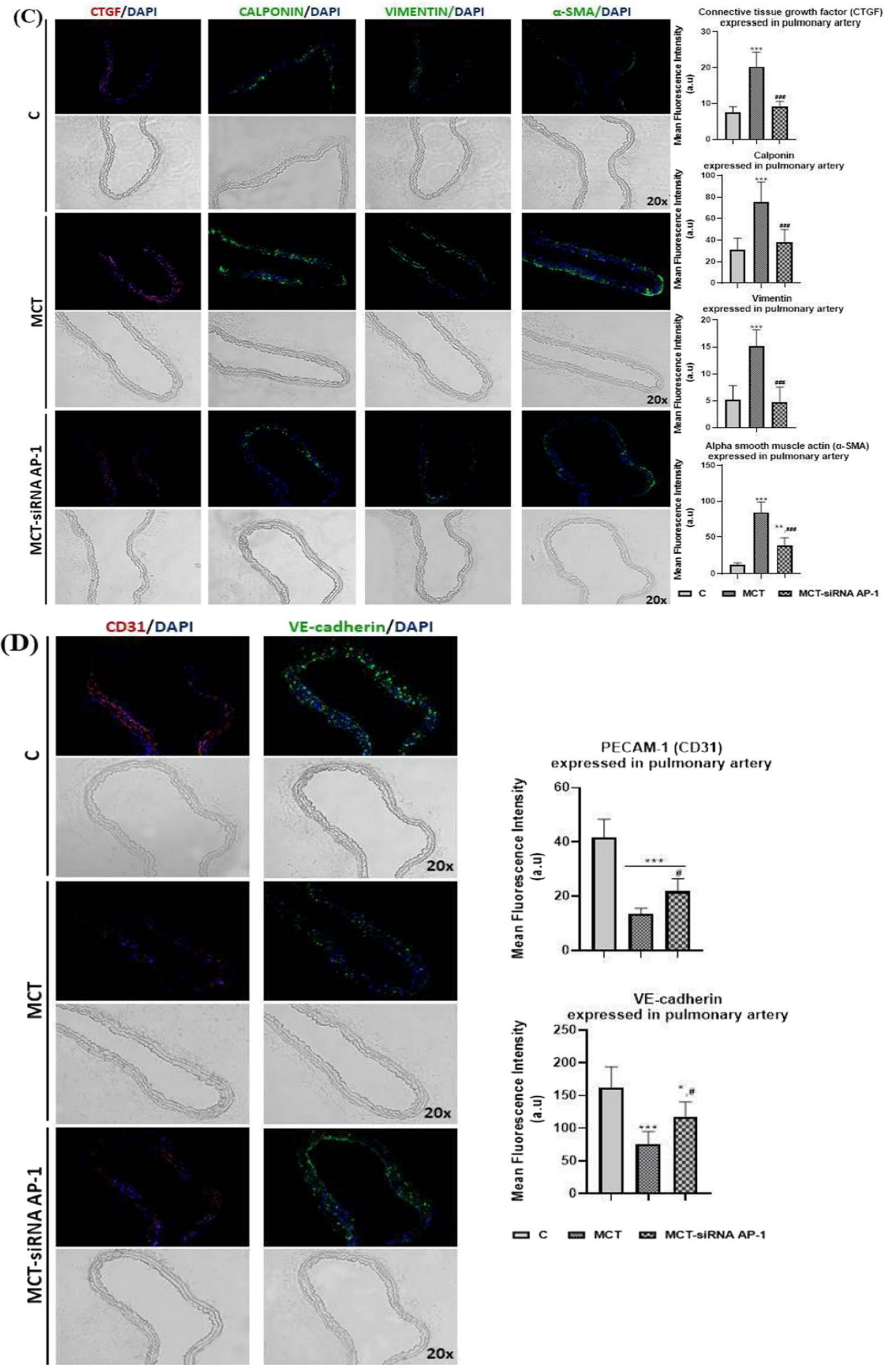

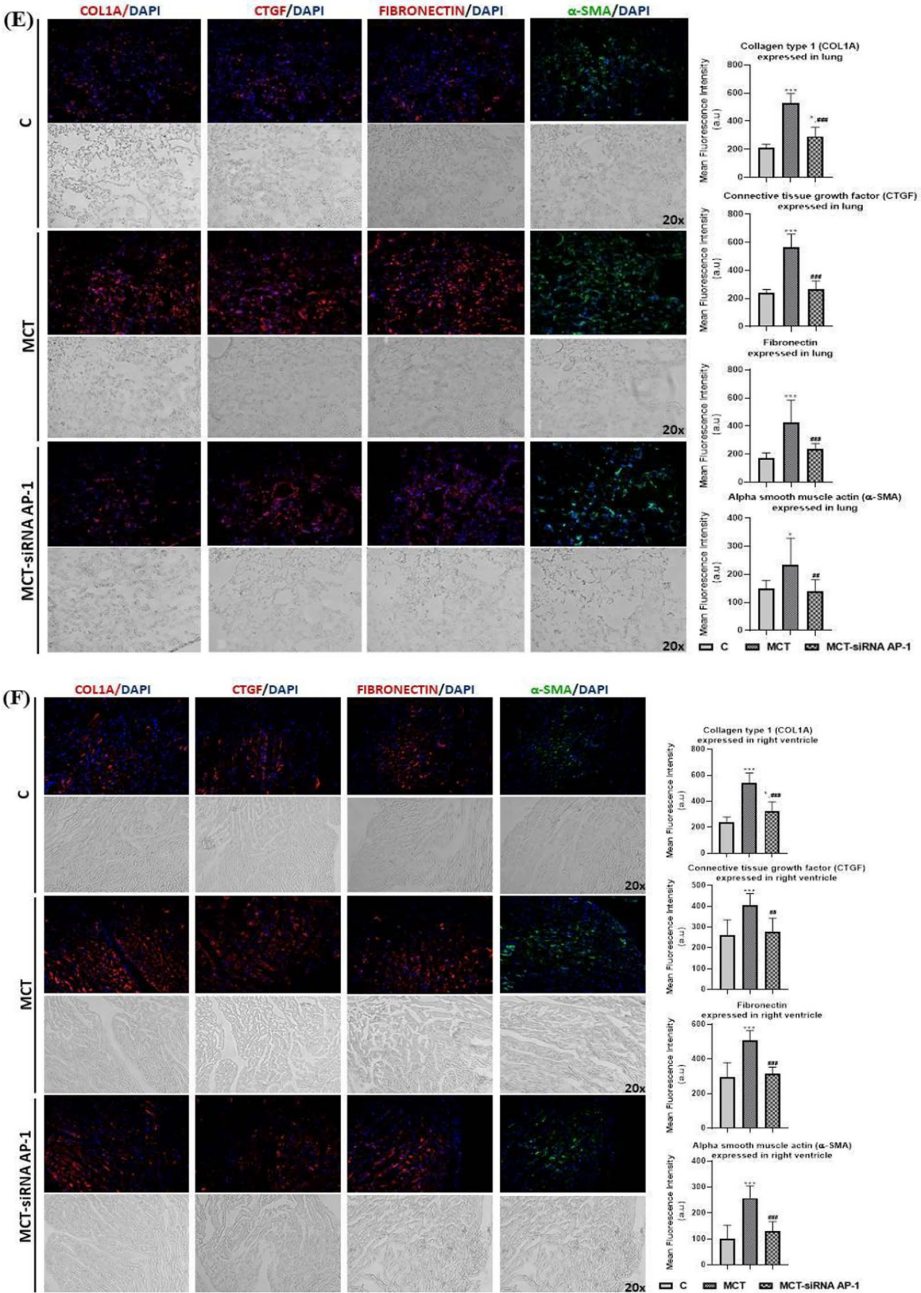

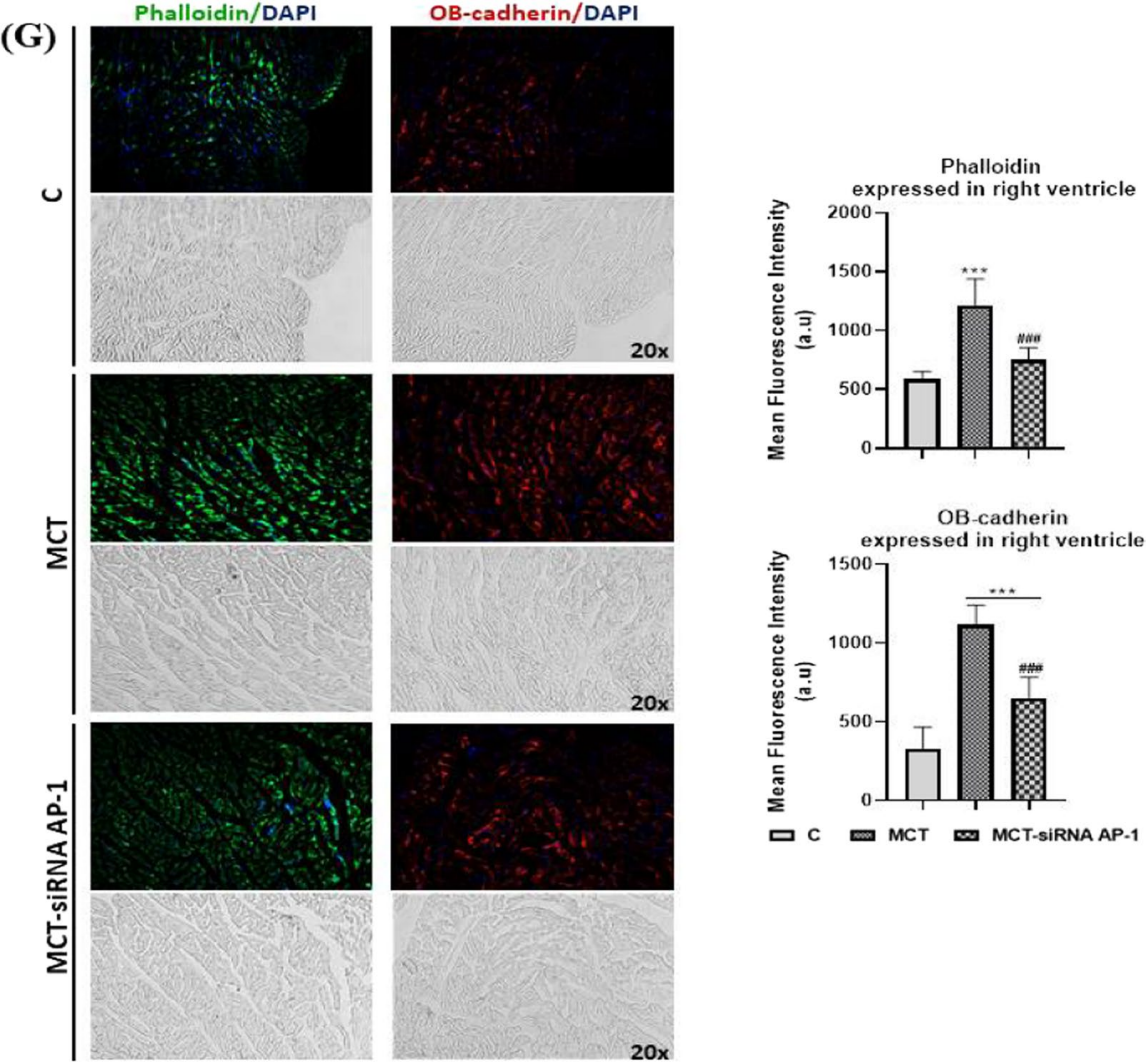
Representative immunofluorescence images for the evaluation of oxidative stress, remodelling pathway, and fibrosis markers specific to vascular dysfunction, lung and cardiac fibrosis after 12 weeks of MCT alone or in combination with siRNA AP-1 for 10 weeks. The thin cryo-sections from three types of tissues: pulmonary artery, lung and right ventricle harvested from all experimental groups (C, MCT, MCT-siRNA AP-1) were immuno-labelled for: **A** Cytosolic ROS (Reactive Oxygen Species) production stained with dihydroethidium (DHE) dye shown in bright red nuclear fluorescence; Bar graph with quantification of the stained areas expressed in: (a) pulmonary artery, (b) lung, (c) right ventricle, (d) liver. **B** proteins involved in vascular remodelling/fibrosis: collagen type I (COL1A1-red), Fibronectin-red, Matrix metalloproteinase-9 (MMP-9-green); Bar graph with quantification of the stained areas expressed in pulmonary artery. **C** proteins involved in vascular fibrosis and the epithelial-mesenchymal transition (EMT): Connective tissue growth factor (CTGF-red), Calponin-red, Vimentin-red, alpha smooth muscle actin (α-SMA-green); Bar graph with quantification of the stained areas expressed in pulmonary artery. **D** specific endothelial markers for EMT assessment: PECAM-1 (CD31-red), Vascular endothelial cadherin (VE-cadherin-green); Bar graph with quantification of the stained areas in pulmonary artery. **E** proteins involved in pulmonary fibrosis and EMT: COL1A1-red, CTGF-red, Fibronectin-red, α-SMA-green; Bar graph with quantification of the stained areas expressed in lung tissue. **F**, **G** proteins involved in cardiac hypertrophy/fibrosis and FMT: COL1A1-red, CTGF-red, Fibronectin-red, α-SMA-red, F-actin (Phalloidin-green) OB-cadherin-red; Bar graph with quantification of the stained areas expressed in right ventricle tissue. Nuclei were stained with DAPI dye shown in blue fluorescence. Data were presented as mean ± SD. Each experiment point was performed in triplicate, from two different set of experiments. Five different microscopic fields for each experimental point were analysed. 20 × magnification, images quantified using the ImageJ program. The statistical significance, noticeably different, was represented as ***P < 0.005, **P < 0.01, *P < 0.05 values versus control group and ^###^P < 0.005, ^##^P < 0.01, ^#^P < 0.05 values versus MCT group. One-way ANOVA, Bonferroni post-test was applied

First of all, the levels of cytosolic ROS (reactive oxygen species) in the pulmonary artery, lung, right ventricle, and liver, were assessed by fluorescence microscopy (Fig. 9A). After analysing the fluorescence images, it was found that the MCT group had considerably higher fluorescence intensity values (***P < 0.005) than the control group both in the wall of the pulmonary artery and in the lung, right ventricle, and liver (Fig. 9A). The AP-1 siRNA treatment considerably attenuated the effects induced by MCT, generating significantly reduced ROS fluorescence intensity levels (^###^P < 0.005) (Fig. 9A).

Later, at the level of the pulmonary artery, lung and right ventricle, a series of proteins involved in vascular remodelling/fibrosis (COL1A1, Fibronectin, MMP-9, CTGF, Calponin, Vimentin, α-SMA), pulmonary and cardiac fibrosis (COL1A1, Fibronectin, CTGF, α-SMA) and EMT (α-SMA, PECAM-1 (CD31), VE-cadherin) were investigated by fluorescence microscopy (Figs. 9B–F). In addition, the expressions of F-actin and OB-cadherin (or cadherin-11) with role in inflammation, fibrosis and FMT were considered at the right ventricle level (Fig. 9G).

Analysis of fluorescence images from the pulmonary artery level revealed that, with the exception of CD31 and VE-cadherin markers, where the fluorescence intensity was significantly reduced (***P < 0.005) (Fig. 9D) all other investigated markers (COL1A1, Fibronectin, MMP-9, CTGF, Calponin, Vimentin, α-SMA) had fluorescence intensities significantly higher (***P < 0.005) in the MCT group when compared to control animals, confirming the presence of EMT and the vascular remodelling process (Fig. 9B, C). The group treated with siRNA AP-1 displayed lower expression for these markers (^###^P < 0.005) compared to the MCT group, values that were comparable to those of the control group (Fig. 9B, C). Regarding the markers CD31 and VE-cadherin, an increase in fluorescence intensity was observed compared to the MCT group (^#^P < 0.05) (Fig. 9D).

To track pulmonary fibrosis and EMT, a panel of markers that included COL1A1, CTGF, Fibronectin and α-SMA was analysed by fluorescence microscopy (Fig. 9E). The fluorescence intensity for all these markers was considerably increased after the injection with MCT (***P < 0.005, *P < 0.05), and maintained within normal limits after treatment with siRNA AP-1 (^###^P < 0.005, ^##^P < 0.01) (Fig. 9E).

The same protein panel was investigated in the case of the right ventricle, where cardiac hypertrophy/fibrosis and FMT were generated following the administration of MCT (Fig. 9F). Two additional markers, F-actin (labelled with phalloidin) and OB-cadherin, were analysed for FMT evaluation (Fig. 9G). Again, in this situation, the results in the expression of fluorescent proteins were significantly augmented (***P < 0.005) in the MCT group, while in the MCT-siRNA AP-1 group, the increases in the expression of these proteins were moderated substantially by the treatment with AP-1 (^###^P < 0.005, ^##^P < 0.01) (Fig. 9F, G).

It can be concluded that the structural and functional changes induced by MCT are supported and confirmed by the increases in markers of oxidative stress, remodelling pathways, and fibrosis in pulmonary artery, lung, and right ventricle. It also highlights how siRNA AP-1 therapy reduced and improved all these parameters.

### The signalling molecules that control the inflammatory response displayed a reduced expression profile after siRNA AP-1 treatment

In our animal model with PAH and associated perivascular and interstitial fibrosis induced by MCT, a series of plasma proinflammatory cytokines (ET-1, TGF-β1, IL-1β, TNF-α) and tissue profibrotic molecules (COL1A1, Fibronectin, MMP-9, CTGF, Calponin, Vimentin, α-SMA) had increased protein expressions. Knowing that increased circulating levels of ET-1 activate the AP-1S3 transcription complex through the MAPkinase pathway, and ET-1 also promotes the differentiation of myofibroblasts by activating focal adhesion kinase (FAK), in our study we envisioned that these signalling pathways and molecules could have an important role in increasing the expression of inflammatory and profibrotic factors.

**Fig. 10:**
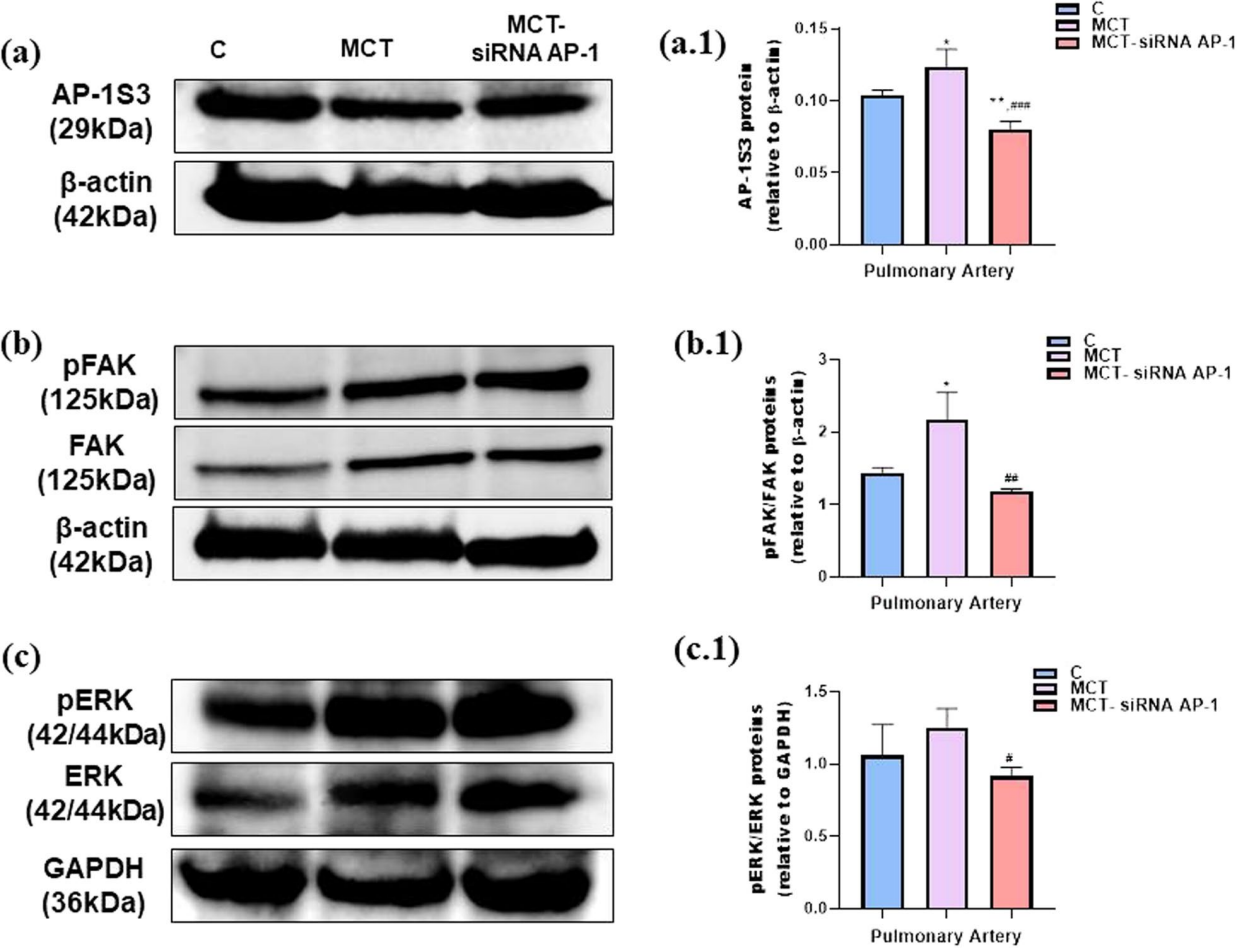
Representative Western blotting images of the expression levels of AP-1S3 (**a**), pFAK, FAK (**b**), pERK, ERK and β-actin/GAPDH (**c**) in pulmonary artery homogenates from all three experimental animal groups after 12 weeks of MCT alone or in combination with siRNA AP-1 for 10 weeks. Histograms show a quantitative representation of signalling molecules, AP-1S3 (**a.1**), pFAK, FAK (**b.1**), pERK, ERK (**c.1**). The data are shown as the mean ± SD of 4 independent experiments. Statistical significance represented as **P < 0.01, *P < 0.05 values versus control group and ^###^P < 0.005, ^##^P < 0.01, ^#^P < 0.05 values versus MCT group, One-way ANOVA, Bonferroni post-test. Quantification of band intensities expressed as ratio with β-actin/GAPDH was analysed by TotalLab TL120 program. The housekeeping β-actin and GAPDH proteins are shown as loading control for protein normalization

According to the findings shown in Fig. 10a.1, a relative increase in the expression of the AP-1 transcription complex could be seen as a result of MCT administration (*P < 0.05). At the same time, treatment based on siRNA AP-1 significantly reduced the levels of this molecule not only when compared to the MCT group (^###^P < 0.005), but also when compared to the control group (**P < 0.01) (Fig. 10a.1). Regarding the FAK protein expression level, a key marker of the fibrotic process, a statistically significant increase was observed in the MCT group (*P < 0.05), which was strongly reduced after 10 weeks of siRNA AP-1 treatment (^##^P < 0.01), the expression level being similar to that recorded in the control group (Fig. 10b.1). Furthermore, the expression level of ERK molecule (member of the “generic” mitogen-activated protein kinase (MAPK) signalling pathway) presented in Fig. 10c.1 increased after the administration of MCT, but without statistical significance. On the other hand, the treatment with siRNA AP-1 considerably reduced the expression of this fibrosis-related protein (^#^P < 0.05) (Fig. 10c.1). The number of animals in each experimental group was 6. In order to obtain a sufficient amount of protein, the arteries from 2 animals from each group were put together.

Consequently, these results demonstrate that the activation of the FAK/ERK/AP-1 signalling pathway and the EMT/FMT transition generates increased expression of proinflammatory/profibrotic markers involved in pulmonary vascular remodelling, pulmonary and cardiac fibrosis, left ventricular hypertrophy and increased blood pressure.

In addition, the inhibition of the AP-1 signalling pathway by siRNA AP-1 treatment, significantly reduced the expression of these signalling molecules, which positively correlates with the reduction of profibrotic and proinflammatory factors and the restoration of certain structural and functional changes previously observed as being generated by the administration of MCT, an inducer of the disease cardiopulmonary.

### Gene expression analysis of a panel of miRNAs involved in the inflammatory response associated with MCT-induced PAH; the effect of siRNA AP-1 treatment

All the structural and functional changes observed in the pulmonary artery, right ventricle and lung, as well as the increased levels of cytokines, proinflammatory and profibrotic markers observed in our study, led us to focus on a panel of miRNAs known to be directly involved in the pathogenesis of PAH [33].

As a result, we assessed the relative gene expression levels of miRNA-124, miRNA-145, miRNA-204, miRNA-210, miRNA-21, and miRNA-214 extracted from pulmonary arteries and right ventricle of the three investigated experimental groups (C, MCT, MCT-siRNA AP-1) in order to complete the table of molecular pathological changes.

**Fig. 11:**
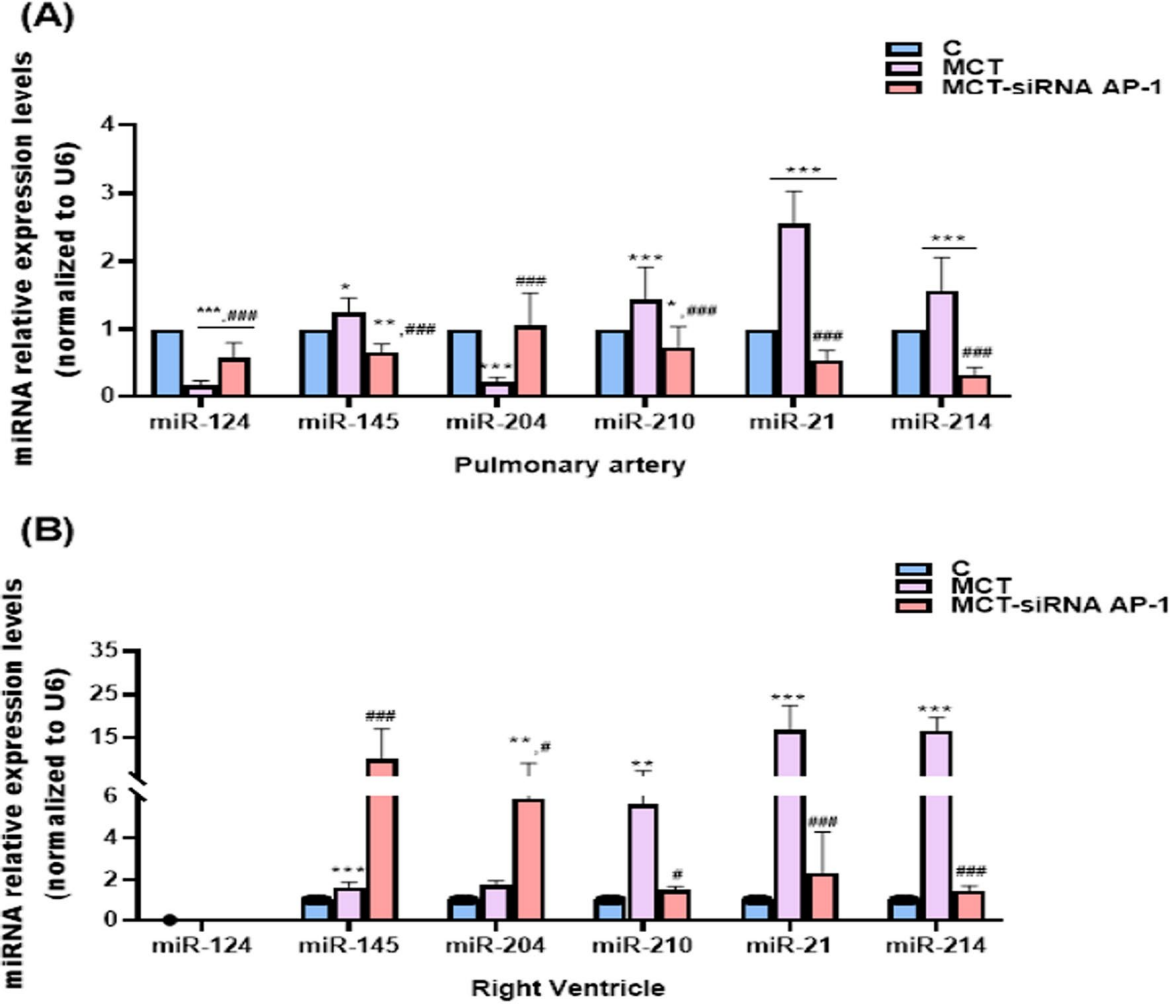
Bar graph that illustrates relative expression levels of six miRNAs, miRNA-124, miRNA-145, miRNA-204, miRNA-210, miRNA-21 and miRNA-214, extracted from **A** pulmonary arteries and **B** right ventricle of the three groups of investigated animals. Total RNA was extracted and used for RT-qPCR assay. The expression of miRNA panel was validated using four tissue samples from each group and matched normal tissue samples. The miRNA expression was normalised using snRU6 as a reference gene. P-values of significant differences between the groups were calculated, and represented as ***P < 0.005, **P < 0.01, *P < 0.05 values versus control group, and ^###^P < 0.005, ^#^P < 0.05 values versus MCT group (One-way ANOVA Bonferroni post-test analysis). The mean fold change in expression of the target miRNA was calculated using ∆∆Ct = ΔCt (a target sample)−ΔCt (a reference sample). For the control sample, ∆∆Ct equals 0 and 2^0^ equals 1, therefore fold change in gene expression relative to control equals 1

Two miRNAs, namely miRNA-124 and miRNA-204, were shown to have down-regulated expressions in the MCT group compared to C group (***P < 0.005) (Fig. 11A). For the other investigated miRNAs, miRNA-145, miRNA-210, miRNA-21, and miRNA-214, the expression levels were significantly increased, they being up-regulated compared to those quantified at hamsters in C group (***P < 0.005,*P < 0.05) (Fig. 11A). The group that received siRNA AP-1 treatment also underwent validation of the expression of this miRNA panel. Thus, according to the findings shown in Fig. 11A, miRNA-124 and miRNA-204 considerably increased (^###^P < 0.005), whereas the expression levels of miRNA-145, miRNA-210, miRNA-21, and miRNA-214 were significantly down-regulated when compared to MCT group (^###^P < 0.005).

In cardiac tissue, specifically in the right ventricle, the same miRNA panel was monitored. The findings displayed in Fig. 11B demonstrated that, in comparison to the C group and the group receiving therapy with siRNA AP-1, the expressions of miRNA-210, miRNA-21, and miRNA-214 were considerably up-regulated in the group receiving MCT (***P < 0.005,*P < 0.05). In the right ventricle, the MCT-siRNA AP-1 group exhibited heightened expression of miRNA-204 (**P < 0.01, ^#^P < 0.05) together with increased expression of miRNA-145 (***P < 0.005, ^###^P < 0.005) in comparison to the MCT group. Regarding miRNA-124, the CT readings derived from ventricular samples remained undetermined.

## Discussion

Pulmonary arterial hypertension (PAH) remains a global health issue, being a complex cardiopulmonary disease, with diverse etiology, difficult to diagnose. One of the major complications of this pathology is represented by right ventricular systolic dysfunction characterized by the transition from adaptive to maladaptive stage of right ventricle hypertrophy to right heart failure being the strongest predictor of mortality in patients with pulmonary hypertension (PH) [34, 35]. The scientific literature presents various animal models for the PH study which aim to understand the pathophysiology/pathogenesis of PH in different stages, but also to test and develop many therapies that would improve the disease prognosis, reducing symptoms and increasing patients' hope and quality of life.

Described for the first time in 1967 by Kay et al. [36], the MCT-induced PH animal model is currently one of the most classic and broadly used in vivo murine models for the PH study because it presents advantages in terms of cost efficiency, simple implementation and reproducibility, but also the presence of histopathological findings similar to those found in human PH such as: endothelial dysfunction, vascular remodelling, excessive proliferation of pulmonary arterial SMCs, hemodynamic changes, inflammation and right ventricular hypertrophy [37, 38]. To have the desired effect of MCT, it is necessary to convert monocrotaline pyrrole (MCTP) to its metabolically active form (dehydromonocrotaline) in the liver under the action of oxidizing processes produced by cytochrome P-450 enzyme CYP3A4 [39]. After MCTP becomes metabolically active, it has a pneumotoxic action causing damage at the level of pulmonary arterial endothelial cells, local pulmonary microvascular thrombosis and endothelial dysfunction resulting in increased pulmonary arterial pressure [40–42].

In the present study, to investigate structural and functional changes as well as molecular mechanisms associated with cardiopulmonary disease, we chose to use the PAH hamster model induced by MCT (60 mg/kg body weight in a single subcutaneous dose) together with the therapeutic approach based on siRNA AP-1 treatment (100 nM administered subcutaneously every 2 weeks), from the early stages of the disease, specifically at two weeks after MCT administration, for a period of 10 weeks. Following the biochemical profile of the animals at 4 and 12 weeks, we found that MCT causes changes in the lipid profile, particularly in the case of triglyceride values that showed significantly higher levels. These findings, which were also reported in other studies, were correlated with hepatic metabolism of this substance [43]. Even though MCT is a toxic substance, the chosen dose concentration should induce the specific changes of this pathology without causing hepatotoxicity or other side effects that would result in the establishment of other comorbidities. It is known from research conducted on numerous experimental animal models, except for the hamster, that MCT at a dose of 20–80 mg/kg body (‘single hit’ or ‘double hit’ approach) [44] has no toxic effect on liver function. Since each species or strain metabolizes MCT differently, in our MCT-induced PAH hamster model, we looked at the hepatic transaminase values throughout the experimental period (4 and 12 weeks) to be sure that the chosen dose of MCT does not induce toxicity, and the model is optimal for PAH study. The results revealed that plasma values of hepatic transaminases remained within normal limits, meaning that the selected dose of 60 mg/kg body for MCT injection had no toxic effect on hamster liver.

Furthermore, we showed that, 12 weeks after administration MCT induced significant increases in systolic and diastolic blood pressure and an increased heart rate, and siRNA AP-1 treatment counteracted the negative effects of MCT on blood pressure. Our findings are supported by other studies that evaluated a series of parameters to monitor the effect of MCT on pulmonary arterial pressure such as: right ventricle end-systolic/end-diastolic pressure, right ventricle end-systolic/end-diastolic volume and heart rate through invasive hemodynamic determinations by recording pressure–volume loops [28, 45–47].

It is known that, once the pulmonary arterial pressure is established, the right ventricle will initially experience adaptive hypertrophy through a compensatory mechanism because the cardiac tissue adapts to the increased workload. Long-term maintenance of an increased cardiac workload, as is the case in our experimental model, causes the right ventricle to progress from a compensatory state to a decompensated right ventricular failure. Accordingly, for the evaluation of all these changes at the level of the pulmonary artery but also for the assessment of the structure and function of the right ventricle, the echocardiographic analysis is absolutely essential [48–51]. Thickening of the inner diameter of the pulmonary artery observed in the case of our model can be correlated with a progressive vascular remodelling accompanied by a perivascular fibrosis but also to arterial stiffening leading to increased systolic blood pressure and consequent cardiac hypertrophy [52]. Arterial stiffening is determined by the decrease in the arterial wall elasticity, also observed in our myography experiments, where the ability to contract and relax to various vasoconstrictor and vasodilator agents of the pulmonary artery was severely affected (decreases pulmonary artery reactivity associated with abnormal vasoconstriction and impaired vasodilation), but also by the increase in Vel analysed by PW Doppler mode, which changes the blood flow hemodynamics leading to damage of peripheral small arteries and microcirculation overall [53]. Turning our attention to the assessment of global right ventricular function, the following key parameters were considered, namely ARV, RVWT, PRVOF and TAPSE which provide important insights into the state of the cardiac tissue, and also serve as a central predictor of survival and prognosis in patients with PAH [54–56]. The reduction of the TAPSE parameter, of the internal cavity of the right ventricle, as well as of the PRVOF, demonstrated that the function of the right ventricle was strongly affected in our MCT-induced PAH hamster model. Treatment with siRNA AP-1 restored the changes in these echocardiographic parameters used to investigate structure and function of the pulmonary artery and right ventricle. Furthermore, our PAH animal model exhibited a series of histopathological alterations in cardiac, lung, and pulmonary vascular tissue also noticed in other studies that included severe chronic human cases of PH [57, 58]. It is important to mention that in our study, siRNA AP-1 treatment counteracted the negative effects of MCT on blood pressure and restored the changes in echocardiographic and histological parameters used to investigate structure and function of the pulmonary artery and right ventricle. Specifically, the administration of siRNA AP-1 in a less advanced stage of PAH significantly attenuated the perivascular fibrosis development at pulmonary artery level, systolic and diastolic blood pressure, but also the vascular wall dysfunction by improving the contraction and relaxation capacity. At the same time, siRNA AP-1 treatment brought improvements in the right ventricular systolic function, right ventricle dilatation, delaying the onset of cardiac hypertrophy.

For a better understanding of the pathophysiological changes associated with PAH, particularly as a result of MCT exposure, optical and electronic microscopy offered critical information on the tissue abnormalities prevalent in the organs of interest investigated in our study. Pulmonary artery endothelium damage determined alveolar septal cell hyperplasia, pulmonary vein occlusion, interstitial fibrosis in the pulmonary parenchyma, but also degeneration of AEC1 and AEC2, as well as interstitial hypercellularity. This may be directly related to the anti-mitotic capabilities of MCT, which generated DNA damage and cell cycle arrest, as well as AEC1 megakaryocytosis and perivascular edema, evidence validated and previously described in another animal model with MCT-induced PAH [59–62]. Studies continue to show the important role of inflammation and immunity in the etiology of PAH. It is well known that ET-1 is a potent endogenous vasoconstrictor produced by endothelial cells. ET-1 not only activates protein kinase C (PKC), which in turn activates transcription factor AP-1 through the MAPkinase pathway, but it also binds to ETA and ETB receptors, causing arterial vasoconstriction. In addition, ET-1, also known as a mitogenic factor for SMCs, facilitates the differentiation of myofibroblasts by activating FAK, being one of the main orchestrator of pulmonary hypertension [63–66]. In addition to these known data, our study showed that, after 4 weeks of MCT administration at PAH hamster model, increases were observed in circulating levels of both ET-1 and proinflammatory cytokines TGF-β1, IL-1β and TNF-α, important mediators of tissue fibrosis, which are responsible not only for the initiation of inflammation at the respiratory level but also for pulmonary arterial remodelling that causes the activation of key molecules involved in the fibrotic process namely AP-1, ERK and FAK. Notably, FAK mediates the transduction of signals from modified adhesion molecules or ET-1/TGF-β1, causing the transcriptional activation of AP-1 that leads to the differentiation of fibroblasts into myofibroblasts [67]. Other proinflammatory cytokines with increased plasma levels, such as IL-1β and TNF-α, secreted mainly by endothelial cells and activated fibroblasts, are responsible for the production of a high level of ROS, which not only maintain inflammation at the microvascular level, but and influence pulmonary vasoreactivity [68].

Furthermore, our results revealed that the immune cell infiltrate in the bronchoalveolar fluid, in particular CD4 and CD8 positive immune cell populations, could maintain an inflammatory microenvironment even after 12 experimental weeks of MCT, aggravating the inflammatory process. Throughout the treatment period with siRNA AP-1, a reduction of inflammatory markers was observed both in the plasma and in the bronchoalveolar fluid.

To have a better picture of the consequences that the activation of certain key regulatory molecules has on the PAH, we focused on investigating specific markers involved in vascular remodelling, pulmonary fibrosis, right ventricular hypertrophy, but also in the epithelial-mesenchymal transition (EMT). EMT is a fundamental process in fibrotic disorders that is controlled by the TGF-β signalling pathway. Endothelial cells can undergo this transition, developing a mesenchymal phenotype, characterized by reduced levels of endothelial markers, VE-cadherin and CD31, and increased levels of typical mesenchymal markers such as calponin, vimentin and α-SMA. The differentiation of lung fibroblasts into activated myofibroblasts has also been shown to be induced by increased soluble profibrotic factors such as ET-1 and TGF-β1 which are responsible for the production and excessive deposition of EMC characterized by high levels of COL1A, fibronectin, MMP-9, α-SMA and CTGF, molecules involved in ECM remodelling, the progression of the fibrotic process as well as in cardiac hypertrophy [69–73].

In our study, EMT was also observed both in the lung and in the cardiac tissue, through the increased expression levels of the markers mentioned above. In addition, the treatment we proposed to block the expression of the transcription factor AP-1 was successful in reducing the inflammatory process by decreasing the levels of proinflammatory cytokines, an effect observed as early as 4 weeks after the initial MCT injection. Moreover, this decrease was correlated with the decrease in the level of oxidative stress but also of proinflammatory and profibrotic markers, inhibiting vascular remodelling, perivascular and interstitial fibrosis, maladaptive cardiac hypertrophy, thus preventing the occurrence of EMT. Numerous studies have noted that oxidative stress becomes evident by the third week of MCT treatment, causing the release of a large amount of endothelium-derived constrictor factors primarily ET-1, which accentuates vascular endothelium damage resulting in ECM remodelling, both at the level arterial as well as in the lung, reduced pulmonary arterial vasodilator response [74], but may also trigger the NRF-2 mediated stress response pathway by increasing caspase-3 activation and release [75] or by activating the NLRP3 inflammasome caused by endothelial cell ferroptosis via HMGB1/TLR4 pathway [76, 77].

In the present study, the molecular mechanisms underlying the appearance of structural and functional changes and inflammatory processes associated with cardiopulmonary disease were also explored. Recent studies indicated that miRNAs may play a crucial role in the genetic regulation and development of the pathological changes that underlie PAH, including the proliferation, migration, activation of endothelial cells, SMCs, and of fibroblasts, as well as the inflammatory processes [33, 78]. Based on these data, we examined the expression of a panel of six miRNAs, including miRNA-124, miRNA-145, miRNA-204, miRNA-210, miRNA-21, and miRNA-214, known to be involved in PAH pathogenesis. It has already been shown that down-regulation of miRNA-124 caused fibroblast hyperproliferation and migration, resulting in progressive adventitial remodelling, as well as in the secretion of proinflammatory cytokines [79, 80] and of miRNA-204, that is primarily expressed in pulmonary artery MSCs, inducing increased resistance to apoptosis, the level being decreased following the release of pro-hypertensive molecules such as ET-1 [81]. Moreover, the increased expression of miRNA-145, labeled as vascular SMC-specific miRNA, coincided with changes in the phenotype of SMCs but also with the increased expression of the markers α-SMA and calponin [82], results also obtained by us on immunolabelled sections from pulmonary artery and right ventricle. Also, the miRNA-210 up-regulation found by us, responsible for HIF-1-dependent pathway activation, is known to have generated resistance to apoptosis and the hyperplasia of vascular SMCs [83]. Our data showed that miRNA-21 and miRNA-214 expressions were also up-regulated in pulmonary artery and right ventricle, miRNA-21 being correlated with suppression of Rho-kinase activity, resulting in decreased angiogenesis and vasodilatation [84], but also proliferation and migration of vascular SMCs [85]. Also, the increased expression of T-cell-derived miRNA-214 was positively correlated with the worsening of perivascular fibrosis mediated by the profibrotic cytokine release that lead to vascular stiffening under conditions of arterial hypertension [86]. It should be noted that, our findings demonstrated that siRNA AP-1 treatment decreased the miRNA-210, miRNA-21, and miRNA-214 expressions in both pulmonary artery and right ventricle.

## Conclusion

These findings established that exposure to MCT increased reactive oxygen species as well as a number of proinflammatory and profibrotic factors, leading to a variety of vascular effects such as: changes in vascular wall architecture, endothelial dysfunction, vascular remodelling, and perivascular fibrosis associated with an increase in pulmonary arterial pressure. Simultaneously, the right ventricle underwent adaptive hypertrophy as a result of compensatory mechanisms adapted to the increased workload, resulting in impaired systolic and diastolic performance. Correspondingly, proposed therapy for cardiopulmonary disease, which uses siRNA to mediate AP-1 transcriptional complex knockdown, yielded promising results with a good safety profile throughout the 12-week trial period, including improved pulmonary arterial and right ventricular function, regression of perivascular and interstitial fibrosis in the pulmonary artery, right ventricle, and lung, and down-regulation of key inflammatory and fibrotic markers. All these observations based on our findings/results make us confident to say that siRNA AP-1- based therapeutic strategy could be promising and feasible for treating patients suffering from PAH in the future.

*Study limitations*: Ideally, the selection of an experimental animal model for researching a condition/disease is based mostly on the reproducibility of pathological features present in humans for which researchers seek an answer. The MCT-induced PAH animal model has been subjected to some controversies in the last fifty years, because its response varies depending on the species, strains, the way the MCT is metabolized, but also the lack of plexiform lesions. Because it is a chemically-induced animal model, it is considered by many to be a toxic model, a high dose of MCT being able to cause serious extra-pulmonary features such as hepatic veno-occlusive disease accompanied by liver failure and portal hypertension, the development of myocarditis, obstructive pulmonary vein thrombosis, which may contribute to higher mortality, which are not associated with severe forms of PAH in humans. As a result, there are still questions whether this model is suitable for preclinical studies of severe plexogenic PAH or is a model of severe angioproliferative PAH. When it comes to practical applications of siRNA-based therapeutics, the possible adverse effects should always be taken into account. It is crucial to note that no potential negative effect of exogenous siRNA AP-1 treatment systemic delivery was identified in our current work. It should also be mentioned that systemic injection can lead to partial degradation of siRNA due to endonucleases and exonucleases present in the body, which could limit the therapeutic effectiveness of this molecule at the targeted tissue level.

## Data Availability

All datasets generated for this study are included in the article.
